# Vibrational Spectra of Nucleotides in the Presence of the Au Cluster Enhancer in MD Simulation of a SERS Sensor †

**DOI:** 10.3390/bios11020037

**Published:** 2021-01-29

**Authors:** Tatiana Zolotoukhina, Momoko Yamada, Shingo Iwakura

**Affiliations:** Department of Mechanical Engineering, University of Toyama, Toyama 930-8555, Japan

**Keywords:** vibrational spectra, molecular dynamics, nucleotides, Au nanoparticle, SERS

## Abstract

Surface-enhanced Raman scattering (SERS) nanoprobes have shown tremendous potential in in vivo imaging. The development of single oligomer resolution in the SERS promotes experiments on DNA and protein identification using SERS as a nanobiosensor. As Raman scanners rely on a multiple spectrum acquisition, faster imaging in real-time is required. SERS weak signal requires averaging of the acquired spectra that erases information on conformation and interaction. To build spectral libraries, the simulation of measurement conditions and conformational variations for the nucleotides relative to enhancer nanostructures would be desirable. In the molecular dynamic (MD) model of a sensing system, we simulate vibrational spectra of the cytosine nucleotide in FF2/FF3 potential in the dynamic interaction with the Au20 nanoparticles (NP) (EAM potential). Fourier transfer of the density of states (DOS) was performed to obtain the spectra of bonds in reaction coordinates for nucleotides at a resolution of 20 to 40 cm^−1^. The Au_20_ was optimized by ab initio density functional theory with generalized gradient approximation (DFT GGA) and relaxed by MD. The optimal localization of nucleotide vs. NP was defined and the spectral modes of both components vs. interaction studied. Bond-dependent spectral maps of nucleotide and NP have shown response to interaction. The marker frequencies of the Au_20_—nucleotide interaction have been evaluated.

## 1. Introduction

Sensing and analysis of DNA sequences and epigenetic structure modifications in the human genome are essential for the understanding of the mechanism of gene expression in cells and the development of diseases. Efforts to detect and map DNA sequences and modifications with 2D materials such as graphene and hexagonal BN have been made and have utilized a number of methods, such as nanopore-based ionic and transverse current, and bonding to complimentary molecules in nanopores and STM. The development of the surface-enhanced and tip-enhanced Raman spectroscopy (surface-enhanced Raman scattering (SERS) and TERS) [1,2,3,4,5] with nanoscale resolution [6,7,8,9] applicable to single biomolecule [10,11] analysis, as well as stimulated Raman scattering and ultrafast two-dimensional infrared (2D-IR) spectroscopy of small organic molecules [11,12,13,14] opens a way to identify molecular structures in a label-free way and investigate mechanisms of interaction with the environment in biomolecules. Attention to DNA molecules in manifold applications, from sequencing, epigenetic studies, disease relation, etc., to nano-biostructures, makes identification of nucleotides and bases in a variety of states highly desirable in the non-contact label-free way of nanophotonic vibrational spectroscopy [15]. SERS is a promising method for nucleic acid [16] and protein detection [17,18]. In terms of its abundant fingerprint information, anti-interference with water, and extraordinarily high sensitivity, SERS holds many attractive advantages over other techniques. Recent research discusses probing of proteins by SERS in solution using gold nanoparticles in the absence of immobilizing agents so that neither the tertiary structure of the proteins nor the surface properties of the nanoparticles are affected [17]. Methods for the rapid and sensitive detection of extended (≥100-base) nucleic acids with reduced preparation are also being developed [16]. They utilize the DNA sequence-specific assembly of silver nanoparticles labelled with a Raman tag to provide molecular recognition of the target DNA with probe orientation and hybridisation procedures found to be critical for the methods.

Another path to the identification of small organic molecules, such as nucleotides, can employ interaction with a nanopore in a 2D solid film, especially graphene [19,20,21,22,23,24,25,26,27], though experiments and calculations with hexagonal BN were also carried out [28,29,30,31]. Detection of the DNA/RNA mononucleotides [32,33,34] and nucleobases [35] with SERS techniques has also been carried out. Despite the many advantages and simplicity of the 2D nanopore sequencing, a few issues [20] remain to be resolved, including controlling the DNA translocation rate, suppressing stochastic nucleobase motions, and determining the signal overlap between different nucleobases. The graphene-based nanopores suffer from significant signal noises due to graphene hydrophobicity and trapping of DNA bases during translocation [36]. The study of the influence of the interaction force between the nucleobases and graphene nanopore on translocating molecules helps to find a solution to some of the mentioned issues.

Surface-enhanced Raman scattering (SERS) using plasmonic metal nanoparticles has been applied to characterize complex biological samples, ranging from biomacromolecules such as nucleic acids and proteins, to living eukaryotic and prokaryotic cells in whole animals for several decades now. The sensitivity of the SERS signal with respect to interactions at the surface of the plasmonic nanoparticles makes it specifically useful for studies of molecular contacts of nanoparticles in biological systems and does not require chemical or biological functionalization.

Control of the 3D structure of DNA and proteins in SERS spectra acquisition still remains a complex question. In the case of DNA molecules, a translocation of the single or double strand through the nanopore substrate has been well established. For proteins, conformation and unfolding are considered. The discrimination of protein conformations is of critical importance for identifying the unfolding states. Attempts to measure dynamic SERS of proteins [37] were carried out over a long acquisition time. Characteristic protein signatures at different time points were observed and compared to conventional Raman with SERS. TG-SERS can distinguish discrete features of proteins, such as the secondary structures, and is therefore indicative of folding or unfolding of the protein [38]. Other studies [39] mention that at a low gold-nanoparticle: protein-molar ratio, significant unfolding of the protein is observed at the surface of the gold NP. When using unfolded proteins, they can be translocated through the nanostructures [40] and nanopores [41]. Therefore, acquisition of SERS spectra of unfolded proteins at the translocation through a nanopore with an attached enhancer NP should be possible in principle. The process of unfolding and conformation should involve the interaction of the building blocks with system elements, as well as between amino acids in chain molecule and tertiary structures. For secondary and tertiary structures, the amino acids closest to the NP can have slight changes in SERS spectra due to the interactions, and simulation of such changes would help to distinguish and register them in acquired spectra of proteins.

**Figure 1:**
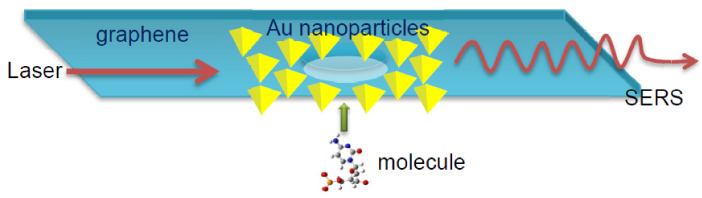
Schematic of the flow-through setup that allows single nucleotide to flow through in a graphene nanopore at plasmonic resonance upon the laser excitation.

In order to design and simulate the system that would allow for single molecule selection and detection of its state by high-resolution SERS methods, we tried to predict direction and possible range of spectral changes in the interacting molecule-SERS enhancer systems. The placement or growth of the small Au nanoparticle at the edge of the graphene nanopore is considered as shown in Figure 1. Plasmonic enhancement by the Au NP will make the vibrational signal measurable, while graphene nanopores would allow for control localization and transient conformation of passing DNA nucleotides, strand fragments, or proteins. Interaction at the Van der Waals interaction distances with both components, graphene and metal NP, has high probability of changing the vibrational spectral maps of the passing molecules. The vibrational mode changes in nucleotides with respect to the interaction with graphene, which were shown in a molecular dynamic (MD) simulation to be present at short distances in our previous calculations. At present, the estimation of the influence of the small Au nanoparticle on the vibrational spectral modes of the cytosine nucleotide has been attempted to be clarified in MD calculations as a model of nucleotide–Au NP interaction. If the spectral modes of both components of interaction are studied relative to localization and molecule conformation, changes in nucleotide spectra due to interaction conditions can be mapped into a kind of library. We performed the initial steps by clarifying the possibility of such mapping. Bond-dependent spectra of nucleotide and NP were tested on their response to interactions to reveal the marker frequencies of the Au_20_—nucleotide interaction.

## 2. Model and Methods

The present study considers the MD approach to the identification of nucleotides in interactions with the nanopore environment for vibrational spectroscopy. The nucleotide vibrational frequencies we obtained have been attributed to stretching, bending, or ring-breathing modes. By comparison of nucleobase spectra to the ones of nucleotides, the presence of the 2′-deoxyribose in the nucleotides can be separated into spectral mapping and mode relaxation. The vibrational density of states is calculated in the transient regime during passing time through the graphene nanopore for each atom of the molecule to resolve the difference in the structure of nucleotides. In dynamics, the vibrational spectra are being evaluated from MD propagation velocities computed in the anharmonic interaction potential, in graphene, and in the molecule, with the Lennard-Jones (LJ) potential between them. Fourier transfer *I(f)* of the velocity autocorrelation function G(*τ*) is as follows:

(1) $$G\left(\tau \right)\;=\;\frac{<v_{i}\left(t_{0}\right)\cdot \;v_{i}\left(t_{0}+\tau \right)>}{<v_{i}\left(t_{0}\right)\cdot \;v_{i}\left(t_{0}\right)>}\;I\left(f\right)\;=\;\overset{\infty }{\underset{-\infty }{\int }}G\left(\tau \right)\exp\left(-2\pi ift\right)d\tau \;$$

where τ is the duration of correlation, $v_{i}\left(t_{0}\right)$ is the velocity of the atom at time $t_{0}$, and $v_{i}\left(t_{0}+\tau \right)$ is the velocity of the atom during correlation time. According to the Wiener–Khinchin theorem, $I\left(f\right)$ defines the vibrational density of states (DOS) of the system. The potential used for DNA nucleotides is the MM2/MM3 force field potential [42]. We investigate the transient interaction with graphene modelled by REBO potential [43] and the molecule-graphene interaction for C-X (X = H, O, C, N) by the LJ potential [42]. The interaction causes a shift in some frequencies of vibrations of the nucleotide DOSs. The vibrational spectra in MD exhibit shifts and intensity changes due to interaction with the environment that causes intramolecular vibrational mode dynamics.

**Figure 2:**
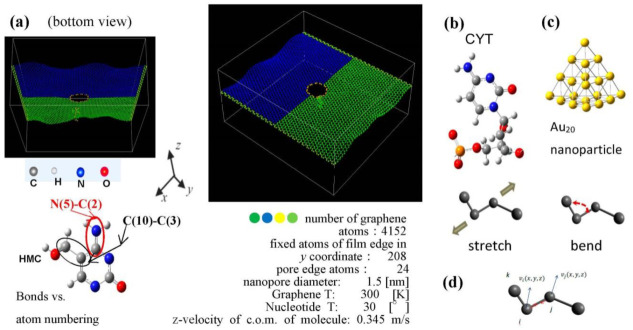
(**a**) The structure and initial position of cytosine nucleotide relative to the graphene pore. The spatial orientation of the nucleotide’s cyclic plane is at 30° to the z-x plane. Atoms are shown: C in grey, N in blue, O in red, and H in light grey. (**b**) Initial configuration of cytosine (right). Hydroxymethylcytosine (HMC) base is shown on the left as an example of corresponding atom numbering for bonds in molecular dynamic (MD) calculations. (**c**) Optimized by density functional theory with generalized gradient approximation (DFT GGA) calculations Au_20_ nanoparticle. (**d**) Reaction coordinates for stretching and bending in velocity correlation calculation use the $\vec{v}$ projections along the bonds.

The calculation setup is presented in Figure 2. The graphene sheet with the 1.5 nm in diameter pore at its center is shown in two projections, a top and front view, with a location of the nucleotide molecule relative to the pore plane and center. The graphene sheet is oriented in the x-y-plane, the edges along the y-axis are fixed, and the edges along the x-axis are free. The nucleotide can move with a given fixed velocity of the center of mass (v_c.o.m._) in the positive z-direction that reproduces the motion of DNA fragments through the pores [44,45] in a driving constant electric field in experimental setups. The v_c.o.m._ is optimized to enhance the interaction force between the nucleotide and edge of graphene pore and reduce rotation of the nucleotide in the translocation as much as possible. All atoms of the system except fixed ones are thermally relaxed to the temperatures T_graphene_ = 300 K and T_nucleotide_ = 30 °C, as in Figure 2, before sampling. In the translocation process, the single-layer graphene sheet interacts with nucleotides in graphene nanopore. Graphene affects the interaction field of the passing molecule close to the pore edge. The graphene-molecule C-X (X = H, O, N, C) potential is considered a VdW one to avoid bond creation and nucleotide attachment to the pore.

The relative size and weight of the nucleotides as compared with nucleobases reduce rotational motion inside the pore [46,47,48,49] that is further controlled by translocation velocity. For the given graphene-nucleotide setup, evaluation of the vibrational spectral maps of the DNA nucleotides, cytosine, thymine, adenine, and guanine, has been initially carried out in reactive coordinates by accumulating spectra of individual atom of bonds obtained by autocorrelation functions of Equation (1). The resulting spectral tables (Tables S1-S8, Figure S1) of each base and nucleotide are included in Supplementary Materials. It was confirmed that the nucleotides–graphene pore interaction does not generate recognizable changes in the spectral map of graphene pore atoms in our system. However, the conformation of the molecules responds to the interaction flexibly, exhibiting spectral changes. The center of mass (c.o.m.) of nucleotides was adjusted relative to the center of the pore along the y-axis to maximize force at the pore edge. The orientation of the cyclic planes of each type of nucleotide was selected to have the same tilt angle for comparison of spectra for various nucleotide interactions. An initial position of the nucleotides was chosen to be outside the interaction region in the z-direction with the tilt of the nucleobase plane taken to be 30 degrees relative to z-x-plane. The single-layer graphene was thermally equilibrated at room temperature prior to sampling. The present simulation was performed in the absence of hydration in the system and hydrophobicity of graphene; therefore, the electrostatic interactions term is not included. For correlation evaluation of nucleotide spectra, the interaction time with graphene was taken at the interaction interval of the passing time through the pore. The high-resolution spectral calculations were carried out at the step size of Δt  = 0.05 fs and τ  = 32,768 steps of correlation delay for the shown states of nucleotides; in the present calculations, cytosine was used to test the system. To attribute obtained frequencies to a particular type of vibrations, such as stretching, bending, or torsion, the autocorrelation function of Equation (1) was calculated over the reaction coordinates that were taken by the projection of velocities on the bond vector, and the respective angle between these vectors as can be seen in Figure 2d. The single frequency obtained in such coordinates can be pinpointed as corresponding to the atom of the vibrating molecule, as well as to the type of vibrational mode that can be pinpointed as corresponding to the particular bond or angle. The ring-breathing modes can be identified by the same frequency present in the spectra of all bonds forming the ring. However, low-level noise from the nearest bonds to the bond studied was also present due to the correlation’s calculation method. Therefore, only relatively high intensity spectral modes were considered for evaluation.

The stability of the obtained frequencies relative to the trajectory randomization has also been tested for cytosine molecules. The 10% to 15% randomization in the initial position and velocity distributions of the cytosine nucleotide at five different calculations have shown a preliminary absence of the changes in spectra above a few percent for stretching of the X-Y (X, Y = C, N, O) bonds. Our calculation system does not include solution molecules yet.

To create a calculation model that would be close to an experimental set up in a SERS type of measurement, we decided to introduce a metal nanoparticle in the nucleotide–graphene system of Figure 1c. The Au_20_ nanoparticle was pyramid-shaped and optimized in ab initio DFT GGA (density functional theory with generalized gradient approximation) calculations with the b3pw91 basis set. The size and shape of the NP was taken to be a stable configuration [50]. The obtained configuration was further relaxed by the MD calculation with the EAM potential [51] and free boundary conditions during 3000 steps with Δt  = 0.1 fs at *T* = 300 K to obtain stable nanoparticle samples that can be used separately or on the top of the graphene sheet. The potential parameters for LJ interaction with nucleotide and graphene were defined using the Lorentz–Berthelot mixing rule and based on the LJ parameters ε  (Au−Au) = 0.039 kcal/mol, σ  (Au−Au) = 2.9 Å [52]. The mixing was done using Van der Waals parameters of the MM2/MM3 force field potential for nucleotides. For the interaction with graphene, mixing used the sp3 C atom parameters from the force field potential, the same values that were applied for the nucleotide–graphene Van der Waals parameters.

**Figure 3:**
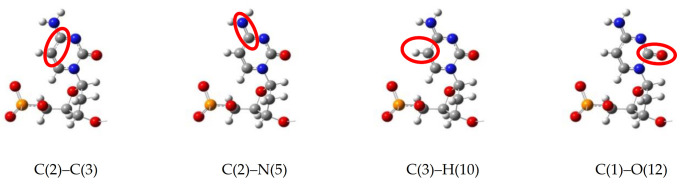
Bonds in cytosine nucleotide (cytidine) for which vibrational spectra are shown below. Numbers of atoms are in internal numbering order.

The study of the vibrational spectra of the nucleotides in the dynamic interaction with metallic nanoparticles (NP) [53,54] located close to the graphene nanopore can combine translocation localization and nucleotide interaction enhancement or modification due to (1) the graphene LJ interaction and (2) the Au LJ interaction. For the initial interaction of the Au_20_ nanoparticle with cytosine nucleotide, the Au_20_ NP was localized close to the cytosine initial position as it is shown in Figure 3. As the first step, both NP and cytosine had zero c.o.m. velocity. Initial orientation and distance of NP relative to the nucleotide was initially controlled to estimate vibrational spectra of both interacting parts of the system.

## 3. Results

The influence of the interaction in the nucleotide-metal nanoparticle system is numerically modelled in the MD system. The validation of the model was performed twofold: first, to obtain a spectral resolution sufficient to register changes due to the LJ interaction, and secondly, to evaluate the corresponding variations in the vibrational spectra of the nucleotide-nanoparticle system.

### 3.1. Resolution of Nucleotide Spectra

First, we evaluated the variations in the nucleotide–graphene system at translocation. Spectral variations which were examined due to interaction force in the nucleobase–graphene nanopore system pointed to the influence of the conformation on the nucleotide spectral maps [55,56]. The transient vibrational frequencies of all passing nucleotides were calculated at the same initial incident angle and shift distance from pore edge. To separate decay in the calculated data from the proper spectrum, we have extracte decay components out of calculated spectral data. The calculation of the transient autocorrelation function includes relatively short interaction time during which the correlation data are accumulated. As a result of the transience of the correlation signal, the exponential function of time decay is converted by the Fourier transfer into a decaying spectral map. In order to separate decay components from the spectra itself, we propose the two-parametric exponential fitting, as follows:

(2) $$x_{\exp fit}\;=\;a\times \exp\left(bx_{1}\right),\;x_{amp\;fit}\;=\;x_{amp}-x_{\exp fit}$$

The introduced exponential function was fitted by parameters a, b  either to the function’s low-frequency region (head) or high-frequency (tail) region. The exponential decay component $x_{exp\;fit}\;$ was then subtracted from the calculated $x_{amp}$ spectra. The low-frequency fitting produced better-resolved spectra above 5 THz compared with the high-frequency fitting. All calculated spectra of nucleotides that are discussed were obtained in the low-frequency fitting. The initial spectral resolution Δf of Fourier transform that was tested was 40 cm^−1^.

In contrast with quantum density functional theory (DFT) calculations of IR and Raman spectra, transient MD calculations are sensitive to the duration of correlation time and relative interaction of the structures studied. Therefore, obtained spectra should be compared with the other available calculations and experimental data. The spectral maps of nucleotides that we calculated were highly sensitive to interactions in the system. Since all nucleotide spectral maps were calculated in identical conditions of nanopore translocation, the obtained transient frequencies could be used as reference values to distinguish nucleotides from each other. Comparison with results of 2D-IR experiments and DFT calculations [57] shows a 50–80 cm^−1^ discrepancy between corresponding stretching C-C and C-N frequencies calculated in the MD model at 40 cm^−1^ resolution. The SERS data on the breathing mode [35] of nucleotides with the full width at half maximum of the peak wavenumber being 13 and 20 cm^−1^ show the presence of the mode in the 660–800 cm^−1^ interval for nucleotides. Our single bond spectra in Appendix A exhibit the presence of the bending type of mode in the above interval, different for each nucleotide; however, our resolution in the case is limited by 40 cm^−1^. For guanine, we obtained a bending peak at 655 cm^−1^ as compared to the measured [35] peak at 661 cm^−1^; thymine exhibited the calculated 860 cm^−1^ peak vs. the measured 795 cm^−1^. All measurements were held in the aqueous solution with nucleotides in proximity to the gold nanoslits or nanoparticles for enhancement of the signal. To improve our model, the resolution was improved and the metal nanoparticle was introduced.

**Figure 4:**
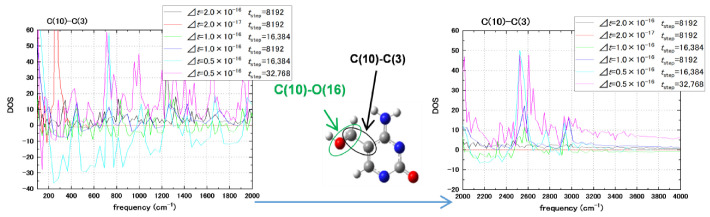
The spectra of a single bond marked as C(10)-C(3) in internal atom numbering of the hydroxymethylcytosine (HMC) nucleotide. The C(10) atom belongs to the hydroxymethyl group and exhibits high frequencies of the C-H bond. The spectra are collected outside of graphene pore interaction (v_z_ = 0) at a different propagation time step (2.0 ÷ 0.5 × 10^−16^ s) and total simulation time (in step number) in two frequency domains: 0–2000 cm^−1^ at the left and 2000–4000cm^−1^ at the right. The base part of the HMC nucleotide with the marked bond adjacent to H-X (C, O) is shown in the center.

The employed spectral resolution in our MD calculations has been sufficient/enough to register structural dependence of the molecular spectral map on a bond-to-bond basis. However, the 2000 cm^−1^ upper limit excludes the C-H and N-H bonds from the calculated frequency region. With the increased resolution of IR and SERS/TERS SM spectroscopy [35,58] and ab initio MD (AIMD) calculations of the chemical bonding effect in SERS [59,60], the information on vibrational mode variations due to interaction with environment, which could be hydration changes or a Van der Waals interaction with graphene pore, should be available in MD calculations as well. The approximation of the MM2/MM3 potential used for nucleotides cannot allow the estimation of dipole moment and polarizability changes with the environment that are calculated in DFT and AIMD spectra. However, transient dynamics sample a sufficient number of states that constitute several periods of vibrations. The coupled anharmonic bonding is also present in the MD potentials which are used. Therefore, a partial reproduction of the changes of dipole moments and polarizabilities with interaction has a cumulative presence in velocity correlation data calculated in MD. To extend the spectral range up to 4000 cm^−1^, we considered the sampling resolution in real space over the interaction potential $U\left(r\left(\Delta t\right)\right)$ by decreasing *∆t,* and in reciprocal space by extending the number of time steps [61]. The testing of the proposed spectral resolution was carried out for the hydroxymethylcytosine (HMC) nucleotide that has H-X bonding sites to X=C and O atoms in hydroxymethyl group (marked in Figure 4).

**Figure 5:**
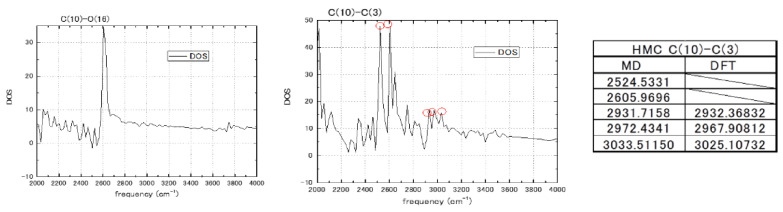
Spectral frequencies of the C(10)-C(3) and C(10)-O(16) bonds of hydroxymethylcytosine (HMC) (see Figure 3) calculated by MD outside of graphene pore (v_z_ = 0) for the 2000–4000 cm^−1^ frequency region. Comparison to the density functional theory (DFT) calculations of Raman frequencies.

As shown in Figure 4, the time step of 0.05 fs and 32,768 steps has given the resolution of vibrational modes at *∆f* = 20 cm^−1^ and up to 4000 cm^−1^. The *∆f* = 20 cm^−1^ spectral resolution is comparable to the 15 cm^−1^ half-width of the Lorentzian function that is used to broaden Raman spectral lines in the DFT calculation [54]. To further confirm our resolution of the Raman spectra of nucleotides, HMC was calculated at the DFT level with the 20 Å size of the supercell for the methylated nucleotide using the Quantum Espresso package [62]. Some of the H-X stretching modes in the hydroxymethylcytosine are shown in Figure 5. The C(10)-C(3) and C(10)-O(16) bonds of hydroxymethyl group are scanned for highest intensity vibration frequencies, which are compared with the Raman frequencies obtained in the DFT calculations. Correspondence, where it was obtained between the MD and DFT frequency modes, is within 10 cm^−1^. Not all MD vibrational modes with high amplitudes correspond to the DFT normal modes. Some of the modes that have high intensity in the MD calculation (2524 and 2605 cm^−1^) can be Raman inactive in the DFT calculation but exhibit IR activity. This relation should be further clarified. The MD calculations with 20 cm^−1^ spectral resolution have been carried out in equilibrium conditions without interaction with graphene.

As compared to the typical sampling timescale of the SERS and TERS techniques being a few microseconds, the typical time scale of the plasmon excitation of the metal enhancer system relates to the scale of 100 fs, and the coupling between the electronic and vibrational degrees of freedom is on the time scale of 0.1–0.5 ps. Therefore, interaction and conformation-dependent single molecule measurements and calculations should concentrate on the time scale of few vibrational periods that we presently cover with a 1.64 ps time interval. Experimentally, nanophotonic probing of the acoustic phonon propagation has been achieved now at the ps timescale [63]. Only the highest peaks in the spectrum are considered as modes in our case. In spectral calculations, the increase in the spectral range resolution simultaneously causes a noise connected to the time step reduction as a consequence of Fourier transfer. The source of the noise is twofold at short time step: (1) the velocity projection on the bond vectors starts to resolve traces of vibrations related to nearest bonds connected by the atom and (2) the FFT procedure in spectra calculations can produce low intensity side lobes of the signal peaks that are not smoothed out with window functions in order not to lose spectral information on essential modes.

### 3.2. Interaction between Metal Clusters and Nucleotides

**Figure 6:**
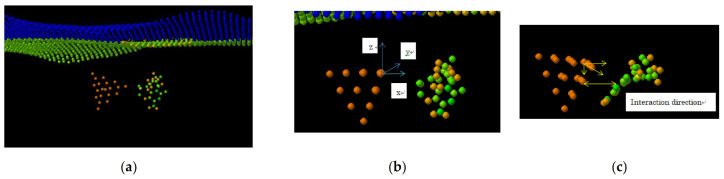
(**a**) System of Au_20_ nanoparticle and cytosine nucleotide vs. graphene sheet in initial Au_20_ NP orientation where interaction is localized primarily within the tip atom of the Au_20_ pyramid; (**b**) rotated NP with the upper plane of the pyramid parallel to the graphene (*x,y*) plane and interaction with the edge atoms of the Au_20_ pyramid enhanced; (**c**) interaction direction of nucleotide with NP changes during nucleotide conformation and alignment at the vibration time. Calculations were first carried out with the initial v_com_ = 0 in x,y,z directions for cytosine and NP.

**Figure 7:**
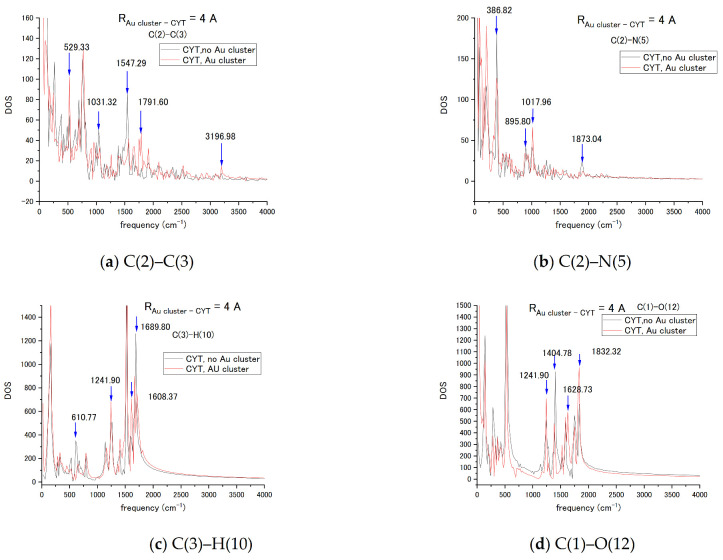
Vibrational spectra of the cytosine (CYT) bonds (**a**) C(2)–C(3), (**b**) C(2)–N(5), (**c**) C(3)–H(10), and (**d**) C(1)–O(12) in the absence and presence of interaction with the Au_20_ NP. Changes in spectra such as mode shifts and large amplitude changes are marked by vertical arrows.

In order to confirm the effect of the LJ interaction on the vibration spectra of in the nucleotide–Au_20_ nanoparticle system, the Au_20_ NP was placed into an already-tested graphene-nucleotide system close to initial nucleotide position as shown in Figure 6a. Cytosine nucleotides’ vibrational spectra have been estimated for base bonds circled in Figure 3 in red. To exclude the interaction with the graphene sheet at the initial stage, the distance from the graphene was out of range of the LJ interaction. The initial orientation of the NP relative to the cytosine nucleotide at the 4 Å distance between the tip of NP and the atoms of the nucleotide was selected to keep the LJ interaction at a relatively weak level. Figure 7 compares vibrational spectra of the bonds C(2)–C(3), C(2)–N(5), C(3)–H(10), and C(1)–O(12) in the absence and presence of Au_20_ NP. Explicit changes in modes due to interaction are shown with vertical arrows ↓ in each case.

In the C(2)–C(3) case, there were changes in bending and twisting at the 500–1000 cm^−1^ frequency range. On the 1300–2000 cm^−1^ interval, the stretch mode is blue shifted due to interaction with NP, so it is attributed to the breathing mode of the nucleotide base. In the C(2)–N(5) bond, the changes in modes related to bending and twisting can be also seen: the amplitude of the breathing mode was very weak, and the C-N stretching mode had no shift but only an increase in amplitude. The C(3)–H(10) bond had changes in C-H bending and stretching modes at the 1000–2000 cm^−1^ interval. For the C(1)–O(12) bond, the largest changes were in breathing and the stretching modes at the 1300–2000 cm^−1^ range. Interaction with the nanoparticle modified transient vibrational frequencies of cytosine at the ps interval in our MD simulation.

**Figure 8:**
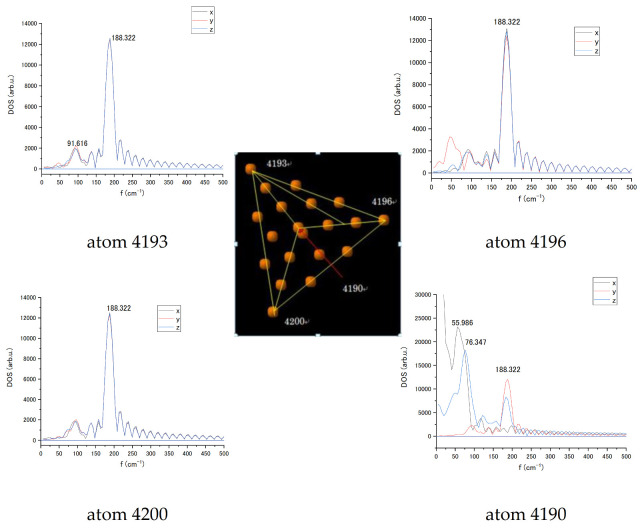
Vibrational spectra of the tip atoms of Au_20_ nanoparticle numbered 4190, 4193, 4196, and 4200 in *x, y*, and *z* coordinates without interaction with the cytosine.

For the Au_20_ NP, the vibrational spectra were also estimated for individual atoms in Cartesian coordinates. Figure 8 exhibits the spectra of the tip atoms in the pyramid in the absence of interaction in x, y, and z coordinates with the basic 188 cm^−^^1^ cluster mode for all tip atoms that can be a mode characterising the whole Au_20_ nanoparticle. Comparing results in Figure 8, it can be seen that the closer to the nucleotide, the stronger the influence. In addition, it can be seen that there is an even greater effect between atoms 4190 and 4196. Obtained spectra relate to a single orientation of the NP vs. nucleotide. A change in NP orientation would lead to variation in LJ interaction strength and subsequent changes in vibration spectrum.

**Figure 9:**
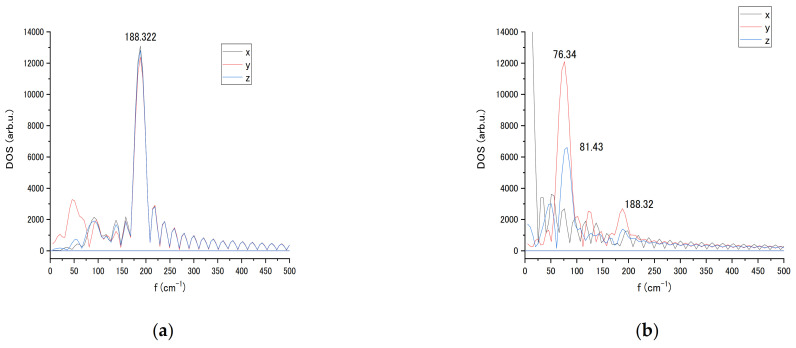
Vibrational spectra of the interacting tip atom (4196) of Au_20_ nanoparticle in x, y, and z coordinates at initial (**a**) and rotated (**b**) orientation relative to cytosine localization.

The Au NP was then rotated as seen in Figure 6b,c. The upper plane of the pyramid was moved to be in the (*x,y*) plane and *z*-axis rotation placed the four atoms of the edge of the pyramid into LJ interaction proximity to the nucleotide. Figure 6c shows the animation snapshot after rotation, and Figure 9 shows the spectral change in 4196 atom’s modes after rotation. The vibrational spectra of the interaction with the cytosine single atom (4196) of Au_20_ nanoparticle tip contrast with the spectra of the rotated NP with edge interaction (Figure 9a,b). The basic 188 cm^−^^1^ frequency of the NP remains intact after rotation. A strong amplitude enhancement is seen for frequencies 76 and 81 cm^−1^ in y and z-direction due to the change in the LJ interaction. The interaction with the nucleotide initiates a slight movement of the NP, which can be reflected in the appearance of the low-frequency modes. For relatively large spherically shaped Au nanoparticles, a typical Raman band over the range of 300–900 cm^−1^ was observed experimentally. For the pyramid-shaped Au_20_ NP, we obtained lower basic frequency that remains stable at different strength of Van der Waals interactions with the nucleotide.

**Figure 10:**
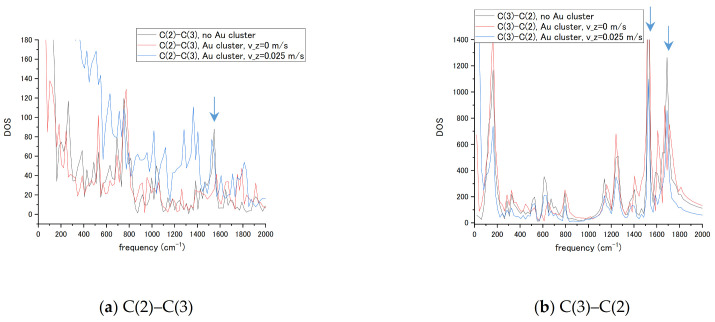
Vibrational spectra of the cytosine bonds (**a**) C(2)–C(3) and (**b**) C(3)–C(2), in the absence and presence of interaction with the Au_20_ NP. Transient velocity of the nucleotide was v_z_ = 0.025 m/s.

The sensor’s design often suggests a fixed attachment of the enhancer nanoparticle and transport of the molecules in the measurement process. To measure transient vibrational spectra of the nucleotides, a scope of molecule’s passing velocity vz has been tested in MD simulations. For the given spectral resolution, a cytosine’s c.o.m. velocity in the range vz = 0.25 m/s = 25 Å/ps has been selected by interaction time being approximately half of the nucleotide’s total propagation time. Passing of the nucleotide limits interaction duration with the NP and leads to changes in vibrational bands. To reveal the connection of the transient time with the changes in the spectrum, we compared spectra of the bond C(2)-C(3) from both end atoms for cytosine alone, a cytosine at 4 Å distance without transient velocity (v_z_ = 0), and with v_z_ = 0.025 m/s as shown in Figure 10. The C(2) atom of the ring is bound to the amino group NH_2_ and C(3) atom of the ring has a C-H bond. The spectra reflect the presence of the different set of bands for each atom in the bond and attachment to the amino group influences transient regime in Figure 10a not only by changes in band amplitude and frequency shifts, but by a velocity of DOS decay that becomes slower. The C(2) atom in Figure 10b exhibits smaller changes in the basic C-C ring vibration frequencies corresponding to ~1550 and 1690 cm^−1^ in present calculations (seen in Figure 7a,c) in stationary and transient regimes. The velocity has been added to the c.o.m. motion of nucleotide at all durations of propagation. Such a simple model reproduces, in general, the motion of partially charged molecules in a uniform electric field. The present result connects the transient velocity of a molecule, size of NP, and interaction time with the molecule’s vibrational spectra. The ring remains relatively stable in the transient regime while the amino group attached to the ring structure responds to the transient interaction.

## 4. Discussion

As seen in existing experimental studies of single-molecule SERS of DNA and proteins [64,65], researchers have gradually developed methods to characterize a single biological molecule with Raman spectra at the time scale of interaction with the environment and other molecules and structures. The simulation methods can create spectral libraries for vibrations of molecular species vs. different types of interaction to specify the interaction’s type and strength.

We connect the importance of the proposed theoretical calculations with the development of machine learning in various fields of biomolecular modeling and simulation. AI algorithms are employed in protein structure prediction based on the data in the form of trajectories that can be used to train machine learning algorithms [66] or applied to the analysis of the SERS/TERS spectra measurements of the presence of drug-in-solution [67]. All such algorithms need data to learn from. Another aspect is that machine learning methods generally require extra preprocessing or knowledge of key features in data of measured structures to handle large-scale data. The proposed simulation approach can be the method to produce a sufficient amount of data to train machine learning algorithms, not only on structural differences in spectra of measured molecules, but also on conformational and interaction changes.

The data on the changes in spectra relative to the interactions in the measured system will provide knowledge of key features in spectra as a part of data sets. Such data in combination with machine learning can reduce spectra accumulation and averages over a few seconds’ acquisition time applied currently, and open the way to a single pulse measurement of vibrational spectra. In the present study, the possibility of the simulation of Raman spectra as depending on the transient interactions between the parts of a SERS system is investigated. A simple system that contains the molecule itself, an Au nanoparticle as Raman enhancer and graphene sheet with nanopore that can control the localization of the measured molecule relative to enhancer if it is attached to graphene, is selected. The position-controlled agglomeration of nanoparticles can be a key to the successful collection of SERS spectra [39] of proteins and DNA as shown in the experiment. We suppose that when the positioning of nanoparticles can be controlled, and detailed knowledge about spectral changes due to interaction with the nanoparticles and substrate such as graphene with nanopore can be used for the training of machine learning algorithms, fast transient single-molecule SERS measurements can be realized.

The question of extension of the achieved accuracy and simulation’ sensitivity of building block molecules to the 3D structures of biomacromolecules such as nucleic acids and proteins are also important for applications. In the case of DNA molecules, a slow translocation of the single or double strand through the nanopore has a timescale around a microsecond in the experiment. The duration time of the nucleotide presence in the vicinity of the graphene nanopore with attached Au NPs should be far below the conventional time resolution of current SERS and TERS techniques that accumulate pulse spectra. However, the single pulse measurement will get a spectrum of the nucleotide located in the vicinity and currently enhanced by agglomerated Au NPs at the translocation time. Each such single pulse spectrum is relatively weak and has a fingerprint of the interaction with the enhancer NP, therefore, preliminary knowledge of the spectral changes relative to interaction could help to isolate the signal in the spectrum from the noise level.

## 5. Conclusions

With the development of machine learning in biomolecular modeling and characterization, knowledge of interaction-dependent features in spectral data of SERS-measured molecules and structures leads to dynamic single-molecule SERS fingerprints. We have shown that the dynamic vibrational motion analysis of a single molecule can investigate the Van der Waals interaction.

The findings presented in the current research prove the MD simulation’s applicability for transient vibrational spectra of biomolecular building blocks such as nucleotides or amino acids. We have shown that the obtained vibrational spectra are sensitive enough to reflect even weak Van der Waals interactions in the few component systems studied, such as nucleotide–Au-NP–graphene. The transient regime of nucleotide passing by NP and through the graphene was shown to be spectrally sensitive. This is confirmed by changes in the bands of amino and methyl groups attached to the rings dependent on interaction strength and length. The transient vibrational spectra record enables discriminating different interaction events with the spectral fingerprints of molecules and NP that also exhibit spectral modification. We consider expanding the scope of the method for protein fragments and conformations. The use of interaction-dependent MD and ab initio MD simulations of transient spectra can make the measurement of SERS of unattached molecules attainable in a small number of pulses.

## Figures and Tables

**Figure A1:**
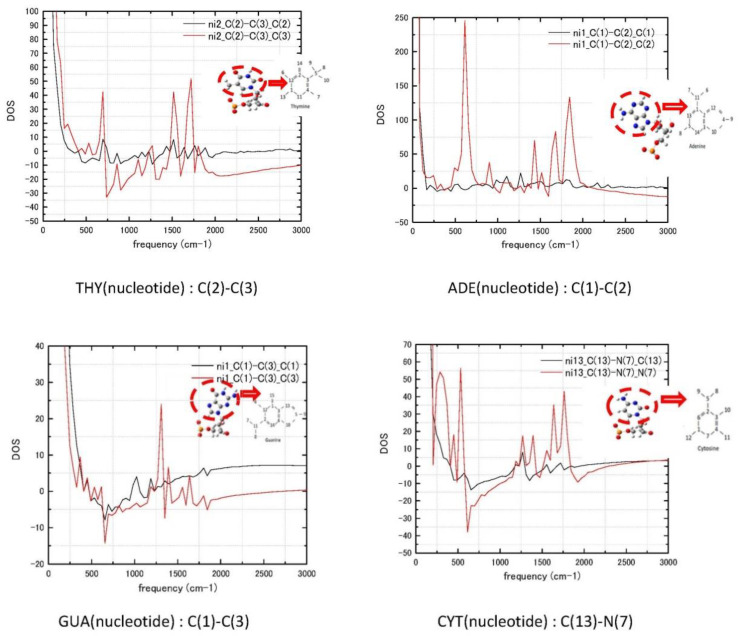
Stretching spectra of adenine, guanine, thymine C(1)–C(2)–C(3) bonds for atoms in hexagonal ring of the base, C(13)–N(7) stretching frequencies of cytosine in the base-to-2′-deoxyribose bond, see numbering in the inserted scheme of each base. Red and black colors mark the *i-j* bond from the *i* and *j* side. Atoms at the side of the bond are written after the bond: X-Y X.

## Data Availability

Not applicable.

## Supplementary Materials

The following are available online at https://www.mdpi.com/2079-6374/11/2/37/s1. Table S1: Frequencies of cytosine nucleotide bonds, highest intensities. Table S2: Frequencies of thymine nucleotide bonds, highest intensities. Table S3: Frequencies of adenine nucleotide bonds, highest intensities. Table S4: Frequencies of guanine nucleotide bonds, highest intensities. Figure S1: The numbering of the nucleotide atoms used in Tables S1–S4 and S5–S8. Table S5: Frequencies of cytosine base bonds, highest intensities. Table S6: Frequencies of thymine base bonds, highest intensities. Table S7: Frequencies of adenine base bonds, highest intensities. Table S8: Frequencies of guanine base bonds, highest intensities.

## Appendix A

For several selected bonds, the stretching spectra of four nucleotides are shown below in Figure A1 for the low resolution calculations.

Each bond between atoms *i* and *j* includes the projection of the vectors ${\bar{v}}_{i}$ and ${\bar{v}}_{j}$ on the bond’s ${\bar{r}}_{ij}$ vector. Investigated spectra for each bond are evaluated from velocity autocorrelations for *i* and *j* atoms and are shown in black and red in the figures. Since each atom participates in several bonds’ vibrations, calculated spectra contain low amplitude bands of adjacent bonds. The valid frequency range is 100–2000 cm^−1^. The calculations of spectra were done for approximately 16 ps, with the time step equal to 0.2 fs, at 40 cm^−1^ resolution. The Tables of calculated vibrational bands for the four types of nucleotides and their bases are collected in Supplementary Materials.
