# Supplementary material for: Vibrational Spectra of Nucleotides in the Presence of the Au Cluster Enhancer in MD Simulation of a SERS Sensor
Source: Biosensors (Basel). 2021 Jan 29;11(2):37. doi: 10.3390/bios11020037 (PMC7911439; doi:10.3390/bios11020037)
Supplement: Supplementary file 1 [file biosensors-11-00037-s001.pdf]

Supplemental material

Biosensors

Vibrational Spectra of Nucleotides in presence of the Au Cluster Enhancer in MD Simulation of a SERS Sensor

Tatiana Zolotoukhina , Momoko Yamada and Shingo Iwakura

Detailed tables of the calculated vibrational frequencies with high intensities for the DNA nucleobases and nucleotides, cytosine, thymine, adenine, and guanine, are collected in the present supporting information. The Green-Kubo method is used to obtain transient spectra in reaction coordinated in the molecular dynamics method.

The spectra of the bonds of atoms in the nucleotides reflect nucleobase structural differences. The spectra of all bonds have been calculated simultaneously at the same translocation through the graphene pore and arranged in the tables presented below. The highest amplitude frequencies of a particular bond are numbered in the sequence of decreasing intensities. They are collected into the spectral maps of molecular species and can be used as a fingerprint for the identification of nucleotides. The first four tables present frequencies for each bond between atoms that have numbers according to the Figs in the text of the paper. Tables 1-4 show results for four nucleobases in the nucleotides. The same frequencies that are present in spectra of many bonds belong to the cyclic ring modes of the particular nucleobase.

The remaining four tables, Tables 5-8, show calculations for the corresponding four nucleobases only, without the presence of the attached 2-deoxyribose.

All calculations of bond stretching in the adjacent tables were performed in the frequency region 100 - 2000 cm^{-1}. The time step was 0.2 fs, calculations of spectra were done for 8192 time steps that correspond to approximately 16 ps duration, and to 40 cm^{-1} resolution. All spectra of nucleotides or bases were obtained from the single MD run. The interaction interval with graphene during transient spectra sampling was sufficient to have intensities of vibrational modes amplified in calculations. The sampling time corresponds to several vibrational periods of bending and stretching modes that bring numerical errors in the computation of frequencies by FFT to a sufficient level.

Table 1 Frequencies of Cytosine nucleotide bonds, highest intensities. Frequencies are in cm^{-1}.

	strech									bend [cm－1]								
塩基名	atom & num	Versus atom	Ref atom	1th peak	2th peak	3th peak	4th peak	5th peak	6th peak	原子. 番号	対原子. 番号	基準原子	第1ピーク	第2ピーク	第3ピーク	第4ピーク	第5ピーク	第6ピーク
CYT	C,1	N,6	C,1	532.6	1188.2	1270.2	1393.1	1638.9		C,1	N,6	C,1	368.8	532.6	614.6	1024.3	1270.2	1638.9
			N,6	532.6	1270.2	1557.0	1638.9					N,6	368.8	573.6	655.6	1188.2	1434.1	
		N,7	C,1	573.6	1188.2	1270.2	1557.0	1679.9	1720.9		N,7	C,1	368.8	532.6	614.6	1024.3	1270.2	1638.9
			N,7	1188.2	1516.0	1557.0						N,7	327.8	573.6	1434.1			
	C,2	C,3	C,2	532.6	1147.2	1393.1	1557.0	1720.9	1843.8	C,2	C,3	C,2	368.8	573.6	614.6	1024.3	1270.2	1638.9
			C,3	1188.2	1557.0	1720.9						C,3	368.8	573.6	655.6	1024.3	1434.1	
		N,5	C,2	450.7	532.6	1393.1					N,5	C,2	368.8	573.6	614.6	1024.3	1270.2	1638.9
			N,5	1188.2	1557.0	1720.9						N,5	368.8	573.6	655.6	1024.3	1434.1	
		N,6	C,2	532.6	1188.2	1270.2	1393.1	1638.9			N,6	C,2	368.8	532.6	614.6	1024.3	1270.2	1638.9
			N,6	1188.2	1270.2	1393.1	1557.0	1720.9				N,6	368.8	573.6	655.6	1024.3	1434.1	
	C,3	C,2	C,3	532.6	1393.1	1557.0	1761.8			C,3	C,2	C,3	368.8	532.6	614.6	1024.3	1270.2	1638.9
			C,2	1188.2	1393.1	1557.0	1720.9					C,2	368.8	573.6	655.6	1024.3	1434.1	
		C,4	C,3	532.6	1393.1	1557.0	1720.9	1843.8			C,4	C,3	368.8	532.6	614.6	1024.3	1270.2	1638.9
			C,4	1188.2	1557.0	1720.9						C,4	368.8	573.6	655.6	1024.3	1434.1	
	C,4	C,3	C,4	532.6	1147.2	1393.1	1557.0	1720.9	1843.8	C,4	C,3	C,4	368.8	532.6	614.6	1024.3	1270.2	1638.9
			C,3	1188.2	1557.0	1720.9						C,3	368.8	573.6	655.6	1024.3	1434.1	
		N,7	C,4	573.6	1188.2	1270.2	1557.0	1720.9	1843.8		N,7	C,4	368.8	532.6	614.6	1024.3	1270.2	1638.9
			N,7	1188.2	1557.0	1720.9						N,7	368.8	573.6	655.6	1024.3	1434.1	
	N,5	C,2	N,5	532.6	1393.1	1557.0	1843.8			N,5	C,2	N,5	368.8	532.6	614.6	1024.3	1270.2	1638.9
			C,2	1188.2	1557.0	1720.9						C,2	368.8	573.6	655.6	1024.3	1434.1	
	N,6	C,1	N,6	532.6	1557.0	1639.0	1720.9			N,6	C,1	N,6	368.8	532.6	614.6	1024.3	1270.2	1638.9
			C,1	532.6	1557.0	1639.0	1720.9					C,1	368.8	573.6	655.6	1024.3	1434.1	
		C,2	N,6	532.6	1393.1	1557.0	1761.8				C,2	N,6	368.8	532.6	614.6	1024.3	1270.2	1638.9
			C,2	532.6	1270.2	1557.0	1639.0	1720.9				C,2	368.8	573.6	655.6	1024.3	1434.1	
	N,7	C,1	N,7	532.6	1270.2	1557.0	1639.0	1720.9	1843.8	N,7	C,1	N,7	368.8	532.6	614.6	1024.3	1270.2	1638.9
			C,1	1188.2	1270.2	1557.0	1639.0					C,1	368.8	573.6	655.6	1024.3	1434.1	
		C,4	N,7	1393.1	1843.8						C,4	N,7	368.8	532.6	614.6	1024.3	1270.2	1638.9
			C,4	1188.2	1270.2	1557.0	1720.9					C,4	368.8	573.6	655.6	1024.3	1434.1	
	H,8	N,5	H,8	450.7	532.6	1393.1				H,8	N,5	H,8	368.8	532.6	614.6	1024.3	1270.2	1638.9
			N,5	1188.2	1270.2	1557.0	1720.9					N,5	368.8	573.6	655.6	1024.3	1434.1	
	H,9	N,5	H,9	450.7	532.6	1393.1				H,9	N,5	H,9	368.8	532.6	614.6	1024.3	1270.2	1638.9
			N,5	1188.2	1270.2	1557.0	1720.9					N,5	368.8	573.6	655.6	1024.3	1434.1	
	H,10	C,3	H,10	532.6	1147.2	1393.1	1557.0	1843.8		H,10	C,3	H,10	368.8	532.6	614.6	1024.3	1270.2	1638.9
			C,3	1188.2	1270.2	1557.0	1720.9					C,3	368.8	573.6	655.6	1024.3	1434.1	
	H,11	C,4	H,11	1393.1	1843.8					H,11	C,4	H,11	368.8	532.6	614.6	1024.3	1270.2	1638.9
			C,4	1188.2	1270.2	1557.0	1720.9					C,4	368.8	573.6	655.6	1024.3	1434.1	
	O,12	C,1	O,12	1270.2	1557.0	1639.0	1720.9			O,12	C,1	O,12	368.8	532.6	614.6	1024.3	1270.2	1638.9
			C,1	450.7	532.6	1270.2	1393.1	1639.0	1720.9			C,1	327.8	573.6	655.6	1024.3	1434.1	

Column names for stretching (left columns) and bending (right columns) frequencies are in cm^{-1} units and as follows:

Base, Atom & num., Bonding atom, Reference atom, 1st mode, 2nd mode, 3rd mode, 4th mode, 5th mode, 6th mode are shown in 2 sets of columns.

Table 2 Frequencies of Thymine nucleotide bonds, highest intensities. Frequencies are in cm-1.

	strech [cm−1]									bend [cm−1]								
塩基名	原子. 番号	対原子. 番号	基準原子	第1ピーク	第2ピーク	第3ピーク	第4ピーク	第5ピーク	第6ピーク	原子. 番号	対原子. 番号	基準原子	第1ピーク	第2ピーク	第3ピーク	第4ピーク	第5ピーク	第6ピーク
THY	C,1	N,11	C,1	409.7	737.5	1024.3	1188.2	1270.2	1679.9	C,1	N,11	C,1	368.8	573.6	655.6	901.4	983.3	1147.2
			N,11	491.7	737.5	1270.2	1516.0	1638.9	1802.8			N,11	327.8	696.5	778.5	860.4		
		N,12	C,1	532.6	655.6	860.4	983.3	1270.2	1516.0		N,12	C,1	368.8	573.6	655.6	901.4	983.3	1147.2
			N,12	532.6	696.5	1270.2	1516.0	1720.9	1802.8			N,12	573.6	696.5	860.4	1024.3	1106.3	
	C,2	C,3	C,2	696.5	1434.1	1516.0	1638.9	1761.8	1884.6	C,2	C,3	C,2	368.8	573.6	655.6	901.4	983.3	1147.2
			C,3	532.6	696.5	1270.2	1516.0	1720.9	1802.8			C,3	573.6	696.5	860.4	1024.3	1106.3	
		N,12	C,2	532.6	655.6	860.4	983.3	1270.2	1516.0		N,12	C,2	368.8	573.6	655.6	901.4	983.3	1147.2
			N,12	532.6	696.5	1270.2	1516.0	1720.9	1802.8			N,12	573.6	696.5	860.4	1024.3	1106.3	
	C,3	C,2	C,3	573.6	860.4	1024.3	1434.1	1679.9	1761.8	C,3	C,2	C,3	368.8	573.6	655.6	901.4	983.3	1147.2
			C,2	532.6	696.5	1270.2	1516.0	1720.9	1802.8			C,2	573.6	696.5	860.4	1024.3	1106.3	
		C,4	C,3	737.5	860.4	1024.3	1434.1	1720.9			C,4	C,3	368.8	573.6	655.6	901.4	983.3	1147.2
			C,4	532.6	696.5	1270.2	1516.0	1720.9	1802.8			C,4	573.6	696.5	860.4	1024.3	1106.3	
		C,5	C,3	696.5	819.5	1024.3	1147.2	1311.1	1516.0		C,5	C,3	368.8	573.6	655.6	901.4	983.3	1147.2
			C,5	532.6	696.5	1270.2	1516.0	1720.9	1802.8			C,5	573.6	696.5	860.4	1024.3	1106.3	
	C,4	C,3	C,4	696.5	1434.1	1516.0	1638.9	1761.8	1884.6	C,4	C,3	C,4	368.8	573.6	655.6	901.4	983.3	1147.2
			C,3	532.6	696.5	1270.2	1516.0	1720.9	1802.8			C,3	573.6	696.5	860.4	1024.3	1106.3	
		N,11	C,4	737.5	1270.2	1679.9					N,11	C,4	368.8	573.6	655.6	901.4	983.3	1147.2
			N,11	532.6	696.5	1270.2	1516.0	1720.9	1802.8			N,11	573.6	696.5	860.4	1024.3	1106.3	
	C,5	C,3	C,5	696.5	1434.1	1516.0	1638.9	1761.8	1884.6	C,5	C,3	C,5	368.8	573.6	655.6	901.4	983.3	1147.2
			C,3	532.6	696.5	1270.2	1516.0	1720.9	1802.8			C,3	573.6	696.5	860.4	1024.3	1106.3	
	H,6	N,12	H,6	532.6	655.6	860.4	983.3	1270.9	1516.0	H,6	N,12	H,6	368.8	573.6	655.6	901.4	983.3	1147.2
			N,12	532.6	696.5	1270.2	1516.0	1720.9	1802.8			N,12	573.6	696.5	860.4	1024.3	1106.3	
	H,7	C,4	H,7	737.5	860.4	1024.3	1434.1	1720.9		H,7	C,4	H,7	368.8	573.6	655.6	901.4	983.3	1147.2
			C,4	532.6	696.5	1270.2	1516.0	1720.9	1802.8			C,4	573.6	696.5	860.4	1024.3	1106.3	
	H,8	C,5	H,8	696.5	819.5	1024.3	1147.2	1311.1	1516.0	H,8	C,5	H,8	368.8	573.6	655.6	901.4	983.3	1147.2
			C,5	532.6	696.5	1270.2	1516.0	1720.9	1802.8			C,5	573.6	696.5	860.4	1024.3	1106.3	
	H,9	C,5	H,9	696.5	819.5	1024.3	1147.2	1311.1	1516.0	H,9	C,5	H,9	368.8	573.6	655.6	901.4	983.3	1147.2
			C,5	532.6	696.5	1270.2	1516.0	1720.9	1802.8			C,5	573.6	696.5	860.4	1024.3	1106.3	
	H,10	C,5	H,10	696.5	819.5	1024.3	1147.2	1311.1	1516.0	H,10	C,5	H,10	368.8	573.6	655.6	901.4	983.3	1147.2
			C,5	532.6	696.5	1270.2	1516.0	1720.9	1802.8			C,5	573.6	696.5	860.4	1024.3	1106.3	
	N,11	C,1	N,11	860.4	1516.0	1720.9	1802.8			N,11	C,1	N,11	368.8	573.6	655.6	901.4	983.3	1147.2
			C,1	491.7	737.5	1270.2	1516.0	1638.9	1802.8			C,1	573.6	696.5	860.4	1024.3	1106.3	
		C,4	N,11	860.4	1516.0	1720.9	1802.8				C,4	N,11	368.8	573.6	655.6	901.4	983.3	1147.2
			C,4	491.7	737.5	1270.2	1516.0	1638.9	1802.8			C,4	573.6	696.5	860.4	1024.3	1106.3	
	N,12	C,1	N,12	860.4	1516.0	1720.9	1802.8			N,12	C,1	N,12	368.8	573.6	655.6	901.4	983.3	1147.2
			C,1	532.6	696.5	1270.2	1516.0	1720.9	1802.8			C,1	573.6	696.5	860.4	1024.3	1106.3	
		C,2	N,12	573.6	860.4	1024.3	1434.1	1679.9	1761.8		C,2	N,12	368.8	573.6	655.6	901.4	983.3	1147.2
			C,2	532.6	696.5	1270.2	1516.0	1720.9	1802.8			C,2	573.6	696.5	860.4	1024.3	1106.3	
	O,13	C,1	O,13	860.4	1229.2	1516.0	1720.9			O,13	C,1	O,13	368.8	573.6	655.6	901.4	983.3	1147.2
			C,1	491.7	737.5	1516.0	1638.9	1802.8				C,1	573.6	696.5	860.4	1024.3	1106.3	
	O,14	C,2	O,14	573.6	860.4	1679.9	1761.8			O,14	C,2	O,14	368.8	573.6	655.6	901.4	983.3	1147.2
			C,2	491.7	737.5	1516.0	1638.9	1802.8				C,2	573.6	696.5	860.4	1024.3	1106.3	

Column names for stretching (left columns) and bending (right columns) frequencies are in cm-1 units and as follows:

Base, Atom & num., Bonding atom, Reference atom, 1st mode, 2nd mode, 3rd mode, 4th mode, 5th mode, 6th mode are shown in 2 sets of columns.

Table 3 Frequencies of Adenine nucleotide bonds, highest intensities. Frequencies are in cm^{-1}.

	strech [cm－1]									bend [cm－1]								
塩基名	原子. 番号	対原子. 番号	基準原子	第1ピーク	第2ピーク	第3ピーク	第4ピーク	第5ピーク	第6ピーク	原子. 番号	対原子. 番号	基準原子	第1ピーク	第2ピーク	第3ピーク	第4ピーク	第5ピーク	第6ピーク
ADE	C,1	C,2	C,1	532.6	983.3	1106.3	1270.2	1802.8		C,1	C,2	C,1	1065.3	1188.2	1311.1	1434.1	1557.0	1761.8
			C,2	614.6	901.4	1434.1	1516.0	1679.9	1843.8			C,2	409.7	614.6	1270.2	1679.9	1884.8	
		N,11	C,1	368.8	614.6	1106.3	1270.2				N,11	C,1	1065.3	1188.2	1311.1	1434.1	1557.0	1761.8
			N,11	532.6	1147.2	1557.0	1638.9	1720.9	1884.8			N,11	860.4	983.3	1065.3	1311.1	1720.9	1802.8
		N,13	C,1	368.8	901.4	1065.3	1188.2	1679.9			N,13	C,1	1065.3	1188.2	1311.1	1434.1	1557.0	1761.8
			N,13	532.6	614.6	901.4	1229.2					N,13	491.7	614.6	696.5	901.4	1475.0	1884.0
	C,2	C,1	C,2	614.6	983.3	1147.2	1229.2	1679.9	1843.8	C,2	C,1	C,2	1065.3	1188.2	1311.1	1434.1	1557.0	1761.8
			C,1	614.6	901.4	1147.2	1434.1	1679.9	1802.8			C,1	491.7	614.6	696.5	901.4	1475.0	1884.0
		C,3	C,2	1106.3	1229.2	1638.9					C,3	C,2	1065.3	1188.2	1311.1	1434.1	1557.0	1761.8
			C,3	614.6	901.4	1147.2	1434.1	1679.9	1802.8			C,3	491.7	614.6	696.5	901.4	1475.0	1884.0
		N,12	C,2	327.8	655.6	1270.2	1638.9	1843.8			N,12	C,2	1065.3	1188.2	1311.1	1434.1	1557.0	1761.8
			N,12	614.6	901.4	1147.2	1434.1	1679.9	1802.8			N,12	491.7	614.6	696.5	901.4	1475.0	1884.0
	C,3	C,2	C,3	532.6	983.3	1106.3	1270.2	1802.8		C,3	C,2	C,3	1065.3	1188.2	1311.1	1434.1	1557.0	1761.8
			C,2	614.6	901.4	1147.2	1434.1	1679.9	1802.8			C,2	491.7	614.6	696.5	901.4	1475.0	1884.0
		N,10	C,3	450.7	655.6	860.4	1229.2	1638.9			N,10	C,3	1065.3	1188.2	1311.1	1434.1	1557.0	1761.8
			N,10	614.6	901.4	1147.2	1434.1	1679.9	1802.8			N,10	491.7	614.6	696.5	901.4	1475.0	1884.0
		N,14	C,3	491.7	778.5	983.3	1106.3	1475.0	1802.8		N,14	C,3	1065.3	1188.2	1311.1	1434.1	1557.0	1761.8
			N,14	614.6	901.4	1147.2	1434.1	1679.9	1802.8			N,14	491.7	614.6	696.5	901.4	1475.0	1884.0
	C,4	N,10	C,4	450.7	655.6	860.4	1229.2	1638.9		C,4	N,10	C,4	1065.3	1188.2	1311.1	1434.1	1557.0	1761.8
			N,10	614.6	901.4	1147.2	1434.1	1679.9	1802.8			N,10	491.7	614.6	696.5	901.4	1475.0	1884.0
		N,12	C,4	327.8	655.6	1270.2	1638.9	1843.8			N,12	C,4	1065.3	1188.2	1311.1	1434.1	1557.0	1761.8
			N,12	614.6	901.4	1147.2	1434.1	1679.9	1802.8			N,12	491.7	614.6	696.5	901.4	1475.0	1884.0
	C,5	N,13	C,5	368.8	901.4	1065.3	1188.2	1679.9		C,5	N,13	C,5	1065.3	1188.2	1311.1	1434.1	1557.0	1761.8
			N,13	614.6	901.4	1147.2	1434.1	1679.9	1802.8			N,13	491.7	614.6	696.5	901.4	1475.0	1884.0
		N,14	C,5	491.7	778.5	983.3	1106.3	1475.0	1802.8		N,14	C,5	1065.3	1188.2	1311.1	1434.1	1557.0	1761.8
			N,14	614.6	901.4	1147.2	1434.1	1679.9	1802.8			N,14	491.7	614.6	696.5	901.4	1475.0	1884.0
	H,6	N,11	H,6	368.8	614.6	1106.3	1270.2			H,6	N,11	H,6	1065.3	1188.2	1311.1	1434.4	1557.0	1761.8
			N,11	614.6	901.4	1147.2	1434.1	1679.9	1802.8			N,11	491.7	614.6	696.5	901.4	1475.0	1884.0
	H,7	N,11	H,7	368.8	614.6	1106.3	1270.2			H,7	N,11	H,7	1065.3	1188.2	1311.1	1434.4	1557.0	1761.8
			N,11	614.6	901.4	1147.2	1434.1	1679.9	1802.8			N,11	491.7	614.6	696.5	901.4	1475.0	1884.0
	H,8	C,5	H,8	327.7	901.4	1270.2	1434.1	1638.9	1761.8	H,8	C,5	H,8	1065.3	1188.2	1311.1	1434.4	1557.0	1761.8
			C,5	614.6	901.4	1147.2	1434.1	1679.9	1802.8			C,5	491.7	614.6	696.5	901.4	1475.0	1884.0
	H,9	C,4	H,9	614.6	819.5	983.3	1147.2	1311.1	1557.0	H,9	C,4	H,9	1065.3	1188.2	1311.1	1434.4	1557.0	1761.8
			C,4	614.6	901.4	1147.2	1434.1	1679.9	1802.8			C,4	491.7	614.6	696.5	901.4	1475.0	1884.0
	N,10	C3	N,10	1106.3	1229.2	1638.9				N,10	C3	N,10	1065.3	1188.2	1311.1	1434.1	1557.0	1761.8
			C,3	614.6	901.4	1147.2	1434.1	1679.9	1802.8			C,3	491.7	614.6	696.5	901.4	1475.0	1884.0
		C,4	N,10	614.6	819.5	983.3	1147.2	1311.1	1557.0		C,4	N,10	1065.3	1188.2	1311.1	1434.1	1557.0	1761.8
			C,4	614.6	901.4	1147.2	1434.1	1679.9	1802.8			C,4	491.7	614.6	696.5	901.4	1475.0	1884.0
	N,11	C,1	N,11	532.6	983.3	1106.3	1270.2	1802.8		N,11	C,1	N,11	1065.3	1188.2	1311.1	1434.1	1557.0	1761.8
			C,1	532.6	1147.2	1557.0	1638.9	1720.9	1884.8			C,1	491.7	614.6	696.5	901.4	1475.0	1884.0
	N,12	C,2	N,12	532.6	983.3	1106.3	1270.2	1802.8		N,12	C,2	N,12	1065.3	1188.2	1311.1	1434.1	1557.0	1761.8
			C,2	532.6	1147.2	1557.0	1638.9	1720.9	1884.8			C,2	491.7	614.6	696.5	901.4	1475.0	1884.0
		C,4	N,12	614.6	819.5	983.3	1147.2	1311.1	1557.0		C,4	N,12	1065.3	1188.2	1311.1	1434.1	1557.0	1761.8
			C,4	532.6	1147.2	1557.0	1638.9	1720.9	1884.8			C,4	491.7	614.6	696.5	901.4	1475.0	1884.0
	N,13	C,1	N,13	532.6	983.3	1106.3	1270.2	1802.8		N,13	C,1	N,13	1065.3	1188.2	1311.1	1434.1	1557.0	1761.8
			C,1	532.6	614.6	901.4	1229.2					C,1	491.7	614.6	696.5	901.4	1475.0	1884.0
		C,5	N,13	860.4	1065.3	1229.2	1597.9	1720.9			C,5	N,13	1065.3	1188.2	1311.1	1434.1	1557.0	1761.8
			C,5	532.6	614.6	901.4	1229.2					C,5	491.7	614.6	696.5	901.4	1475.0	1884.0
	N,14	C,3	N,14	1106.3	1229.2	1638.9				N,14	C,3	N,14	1065.3	1188.2	1311.1	1434.1	1557.0	1761.8
			C,3	532.6	614.6	901.4	1229.2					C,3	491.7	614.6	696.5	901.4	1475.0	1884.0
		C,5	N,14	860.4	1065.3	1229.2	1597.9	1720.9			C,5	N,14	1065.3	1188.2	1311.1	1434.1	1557.0	1761.8
			C,5	532.6	614.6	901.4	1229.2					C,5	491.7	614.6	696.5	901.4	1475.0	1884.0

Column names for stretching (left columns) and bending (right columns) frequencies are in cm^{-1} units and as follows:

Base, Atom & num., Bonding atom, Reference atom, 1st mode, 2nd mode, 3rd mode, 4th mode, 5th mode, 6th mode are shown in 2 sets of columns.

Table 4 Frequencies of Guanine nucleotide bonds, highest intensities. Frequencies are in cm^{-1}.

	strech								
base	atom & num	Versus atom	Ref atom	1th peak	2th peak	3th peak	4th peak	5th peak	6th peak
GUA	C,1	C,3	C,1	696.5	1024.3	1188.2	1352.1	1802.8	
			C,3	368.8	450.7	1188.2	1311.1	1393.1	1638.9
		N,12	C,1	532.6	819.5	901.4	1557.0		
			N,12	573.6	1188.2	1311.1	1393.1	1557.0	1638.9
	C,2	N,11	C,2	450.7	614.6	1352.1			
			N,11	573.6	1188.2	1311.1	1393.1	1557.0	
		N,12	C,2	532.6	819.5	901.4	1557.0		
			N,12	573.6	1188.2	1311.1	1393.1	1557.0	1638.9
		N,14	C,2	450.7	1024.3	1638.9			
			N,14	573.6	1188.2	1311.1	1393.1	1557.0	1638.9
	C,3	C,1	C,3	450.7	1311.1	1557.0	1638.9	1802.8	
			C,1	368.8	450.7	1188.2	1311.1	1393.1	1638.9
		C,4	C,3	532.6	1024.3	1393.1	1557.0	1638.9	1720.9
			C,4	368.8	450.7	1188.2	1311.1	1393.1	1638.9
		N,13	C,3	409.7	737.5	1188.2	1393.1	1720.9	
			N,13	368.8	450.7	1188.2	1311.1	1393.1	1638.9
	C,4	C,3	C,4	696.5	1024.3	1188.2	1352.1	1802.8	
			C,3	368.8	450.7	1188.2	1311.1	1393.1	1638.9
		N,10	C,4	532.6	1188.2	1311.1	1557.0	1720.9	
			N,10	368.8	450.7	1188.2	1311.1	1393.1	1638.9
		N,14	C,4	450.7	532.6	1024.3	1188.2	1638.9	
			N,14	368.8	450.7	1188.2	1311.1	1393.1	1638.9
	C,5	N,10	C,5	532.6	1188.2	1311.1	1557.0	1720.9	
			N,10	368.8	450.7	1188.2	1311.1	1393.1	1638.9
		N,13	C,5	409.7	737.5	1188.2	1393.1	1720.9	
			N,13	368.8	450.7	1188.2	1311.1	1393.1	1638.9
	H,6	N,12	H,6	532.6	614.6	819.5	901.4	1557.0	
			N,12	368.8	450.7	1188.2	1311.1	1393.1	1638.9
	H,7	N,11	H,7	450.7	614.6	860.4	942.3		
			N,11	368.8	450.7	1188.2	1311.1	1393.1	1638.9
	H,8	N,11	H,8	450.7	614.6	860.4	942.3		
			N,11	368.8	450.7	1188.2	1311.1	1393.1	1638.9
	H,9	C,5	H,9	737.5	901.4	1270.2	1393.1	1720.9	
			C,5	368.8	450.7	1188.2	1311.1	1393.1	1638.9
	N,10	C,4	N,10	532.6	1024.3	1393.1	1557.0	1638.9	1720.9
			C,4	368.8	450.7	1188.2	1311.1	1393.1	1638.9
		C,5	N,10	737.5	901.4	1270.2	1393.1	1720.9	
			C,5	368.8	450.7	1188.2	1311.1	1393.1	1638.9
	N,11	C,2	N,11	532.6	655.6	1557.0	1638.9	1720.9	
			C,2	368.8	450.7	1188.2	1311.1	1393.1	1638.9
	N,12	C,1	N,12	450.7	737.5	1311.1	1638.9	1802.8	
			C,1	573.6	1188.2	1311.1	1393.1	1557.0	1638.9
		C,2	N,12	532.6	696.5	1557.0	1638.9		
			C,2	573.6	1188.2	1311.1	1393.1	1557.0	1638.9
	N,13	C,3	N,13	450.7	696.5	1024.3	1229.2	1802.8	
			C,3	573.6	1188.2	1311.1	1393.1	1557.0	1638.9
		C,5	N,13	901.4	1270.2	1393.1	1720.9		
			C,5	573.6	1188.2	1311.1	1393.1	1557.0	1638.9
	N,14	C,2	N,14	532.6	696.5	1557.0	1638.9		
			C,2	573.6	1188.2	1311.1	1393.1	1557.0	1638.9
		C,4	N,14	532.6	1024.3	1393.1	1557.0	1638.9	1720.9
			C,4	573.6	1188.2	1311.1	1393.1	1557.0	1638.9
	O,15	C,1	O,15	450.7	1311.1	1557.0	1638.9	1802.8	
			C,1	409.7	491.7	655.6	1311.1	1679.9	1843.8

bend								
base	atom & num	Versus atom	Ref atom	1th peak	2th peak	3th peak	4th peak	5th peak
C,1	C,3	C,1	245.8	532.6	1106.3	1188.2	1352.1	1638.9
		C,3	163.9	409.7	696.5	1024.3	1311.1	
	N,12	C,1	245.8	532.6	1106.3	1188.2	1352.1	1638.9
		N,12	286.8	368.8	573.6	655.6	1065.3	1188.2
C,2	N,11	C,2	245.8	532.6	1106.3	1188.2	1352.1	1638.9
		N,11	286.8	368.8	573.6	655.6	1065.3	1188.2
	N,12	C,2	245.8	532.6	1106.3	1188.2	1352.1	1638.9
		N,12	286.8	368.8	573.6	655.6	1065.3	1188.2
	N,14	C,2	245.8	532.6	1106.3	1188.2	1352.1	1638.9
		N,14	286.8	368.8	573.6	655.6	1065.3	1188.2
C,3	C,1	C,3	245.8	532.6	1106.3	1188.2	1352.1	1638.9
		C,1	286.8	368.8	573.6	655.6	1065.3	1188.2
	C,4	C,3	245.8	532.6	1106.3	1188.2	1352.1	1638.9
		C,4	286.8	368.8	573.6	655.6	1065.3	1188.2
	N,13	C,3	245.8	532.6	1106.3	1188.2	1352.1	1638.9
		N,13	286.8	368.8	573.6	655.6	1065.3	1188.2
C,4	C,3	C,4	245.8	532.6	1106.3	1188.2	1352.1	1638.9
		C,3	286.8	368.8	573.6	655.6	1065.3	1188.2
	N,10	C,4	245.8	532.6	1106.3	1188.2	1352.1	1638.9
		N,10	286.8	368.8	573.6	655.6	1065.3	1188.2
	N,14	C,4	245.8	532.6	1106.3	1188.2	1352.1	1638.9
		N,14	286.8	368.8	573.6	655.6	1065.3	1188.2
C,5	N,10	C,5	245.8	532.6	1106.3	1188.2	1352.1	1638.9
		N,10	286.8	368.8	573.6	655.6	1065.3	1188.2
	N,13	C,5	245.8	532.6	1106.3	1188.2	1352.1	1638.9
		N,13	286.8	368.8	573.6	655.6	1065.3	1188.2
H,6	N,12	H,6	245.8	532.6	1106.3	1188.2	1352.1	1638.9
		N,12	286.8	368.8	573.6	655.6	1065.3	1188.2
H,7	N,11	H,7	245.8	532.6	1106.3	1188.2	1352.1	1638.9
		N,11	286.8	368.8	573.6	655.6	1065.3	1188.2
H,8	N,11	H,8	245.8	532.6	1106.3	1188.2	1352.1	1638.9
		N,11	286.8	368.8	573.6	655.6	1065.3	1188.2
H,9	C,5	H,9	245.8	532.6	1106.3	1188.2	1352.1	1638.9
		C,5	286.8	368.8	573.6	655.6	1065.3	1188.2
N,10	C,4	N,10	245.8	532.6	1106.3	1188.2	1352.1	1638.9
		C,4	286.8	368.8	573.6	655.6	1065.3	1188.2
	C,5	N,10	245.8	532.6	1106.3	1188.2	1352.1	1638.9
		C,5	286.8	368.8	573.6	655.6	1065.3	1188.2
N,11	C,2	N,11	245.8	532.6	1106.3	1188.2	1352.1	1638.9
		C,2	286.8	368.8	573.6	655.6	1065.3	1188.2
N,12	C,1	N,12	245.8	532.6	1106.3	1188.2	1352.1	1638.9
		C,1	286.8	368.8	573.6	655.6	1065.3	1188.2
	C,2	N,12	245.8	532.6	1106.3	1188.2	1352.1	1638.9
		C,2	286.8	368.8	573.6	655.6	1065.3	1188.2
N,13	C,3	N,13	245.8	532.6	1106.3	1188.2	1352.1	1638.9
		C,3	286.8	368.8	573.6	655.6	1065.3	1188.2
	C,5	N,13	245.8	532.6	1106.3	1188.2	1352.1	1638.9
		C,5	286.8	368.8	573.6	655.6	1065.3	1188.2
N,14	C,2	N,14	245.8	532.6	1106.3	1188.2	1352.1	1638.9
		C,2	286.8	368.8	573.6	655.6	1065.3	1188.2
	C,4	N,14	245.8	532.6	1106.3	1188.2	1352.1	1638.9
		C,4	286.8	368.8	573.6	655.6	1065.3	1188.2
O,15	C,1	O,15	245.8	532.6	1106.3	1188.2	1352.1	1638.9
		C,1	286.8	368.8	573.6	655.6	1065.3	1188.2

Column names for stretching (left columns) and bending (right columns) frequencies are in cm^{-1} units and as follows:

Base, Atom & num., Bonding atom, Reference atom, 1st mode, 2nd mode, 3rd mode, 4th mode, 5th mode, 6th mode are shown in 2 sets of columns.

The numbering of the nucleotide atoms used in tables 1~4 and 5-8. Base atoms order is shown for each base, deoxyribose numbering is done consecutively after each base numbering as shown below for cytosine example.

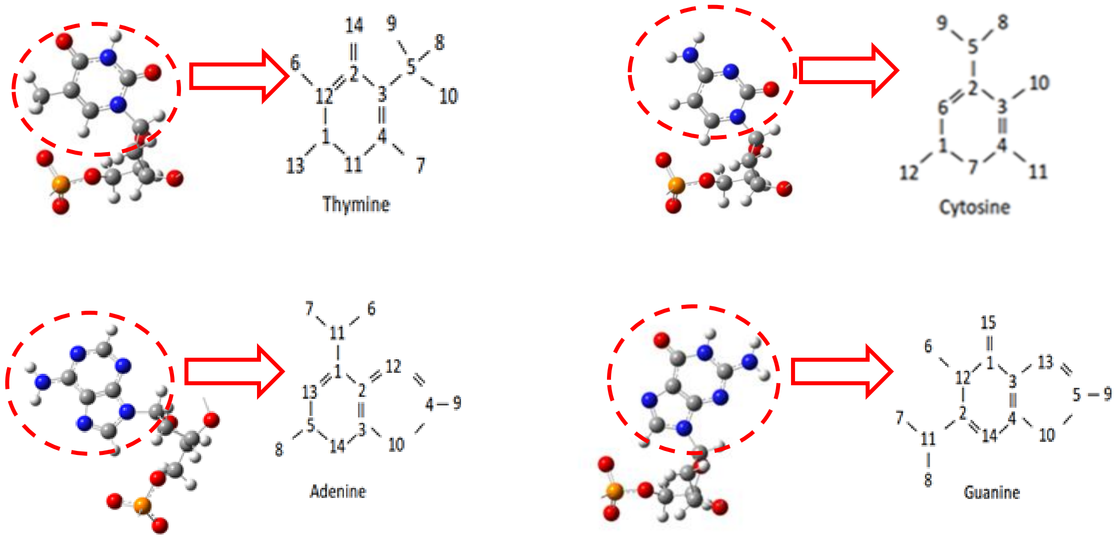

deoxyribose numbering

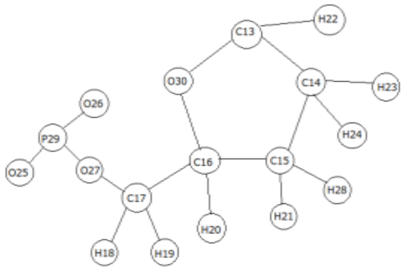

Comparison of calculated frequencies with experimentally measured IR spectra

(C. S. Peng, K. C. Jones, and A. Tokmakoff, "Anharmonic Vibrational Modes of Nucleic Acid Bases Revealed by 2D IR Spectroscopy," J. Am. Chem. Soc., 2011, **133**, 15650-15660)

・CYT

CMP	C1	1651	9.2	1.0	1690	$\nu\,(C^2{=}O)$, $\nu\,(N^1{=}C^6)$, $\delta\,(C^6{-}H)$
	C2	1614	15	0.55	1624	$\nu\,(N^3{=}C^4{-}C^5{=}C^6)$, $\nu\,(C^2{=}O)$, $\delta\,(C^5{-}H)$, $\delta\,(C^6{-}H)$
	C3	1583	17	0.21		
	C4	1524	6.1	0.57	1503	$\nu\,(C^4{-}C^5)$, $\nu\,(N^1{-}C^6)$, $\delta\,(C^5{-}H)$, $\delta\,(C^6{-}H)$, $\delta\,(N^4D_2)$
	C5	1504	8.9	0.94	1483	$\nu\,(N^3{=}C^4{-}N^4)$, $\nu\,(C^5{=}C^6)$, $\delta\,(C^5{-}H)$, $\delta\,(C^6{-}H)$, $\delta\,(N^4D_2)$

Calculated	Experimental		
1721cm^{-1}(C-C , C-N)	1624cm^{-1} →	93cm^{-1}	difference
1557cm^{-1}(C-N)	1503cm^{-1} →	54cm^{-1}	difference

・THY

TMP	T1	1690	11	0.74	1677	$\nu\,(C^2{=}O)$, $\nu\,(C^4{=}O)$, $\delta\,(N^3{-}D)$
	T2	1663	5.0	1.0	1657	$\nu\,(C^4{=}O)$, $\nu\,(C^5{=}C^6)$, $\delta\,(N^3{-}D)$, $\delta\,(C^5H_3)$, $\delta\,(C^6{-}H)$
	T3	1629	10	0.94	1627	$\nu\,(C^5{=}C^6)$, $\nu\,(C^4{=}O)$, $\delta\,(C^5H_3)$, $\delta\,(C^6{-}H)$

Calculated	Experimental		
1721cm^{-1}(C-C , N-C)	1657cm^{-1} →	64cm^{-1}	difference
1762cm^{-1}(N-C)	1677cm^{-1} →	85cm^{-1}	difference

・ADE

AMP	A1	1625	8.0	1.0	1588	$\nu\,(C^4{=}C^5$, $C^5{-}C^6$ out-of-phase), $\delta\,(C^2{-}H)$, $\delta\,(N^6D_2)$, Py
	A2	1578	9.8	0.41	1565	$\nu\,(C^4{=}C^5$, $C^5{-}C^6$ in-phase), $\nu\,(N^1{-}C^6)$, $\nu\,(N^3{-}C^4)$, $\nu\,(N^7{=}C^8)$, $\delta\,(C^8{-}H)$, Py + Im

本 Calculated	Experimental		
1639cm^{-1}(C-C , C-N , N-C)	1588cm^{-1} →	51cm^{-1}	difference

・GUA

GMP	G1	1665	11	1.0	1692	$\nu\,(C^6{=}O)$, $\delta\,(N^1{-}D)$, Py
	G2	1579	4.0	0.63	1556	$\nu\,(C^2{=}N^3)$, $\nu\,(C^6{=}O)$, $\delta\,(N^2{-}H)$, $\delta\,(N^2D_2)$, Py
	G3	1565	7.8	0.62	1533	$\nu\,(C^2{=}N^3{-}C^4{=}C^5)$, $\delta\,(C^8{-}H)$, Py + Im
	G4	1539	18	0.29	1511	$\nu\,(C^4{=}C^5)$, $\nu\,(N^7{=}C^8)$, $\delta\,(C^8{-}H)$, Py + Im

Calculated	Experimental		
1639cm^{-1}(C-N , N-C)	1692cm^{-1} →	54cm^{-1}	difference
1557cm^{-1}(C-C , N-C)	1556cm^{-1} →	1cm^{-1}	difference

Table 5. Frequencies of Cytosine base bonds, highest intensities. Frequencies are in cm^{-1} .

Reaction Coordinate unit of [cm−1]																		
	strech									bend								
base	atom & num	Versus atom	Ref atom	1th peak	2th peak	3th peak	4th peak	5th peak	6th peak	base	atom & num	Versus atom	Ref atom	1th peak	2th peak	3th peak	4th peak	5th peak
CYT	C,1	N,6	C,1	532.6	942.4	1188.2	1516.0			C,1	N,6	C,1	81.9	573.6	1638.9	1761.8		
			N,6	573.6	1557.0	1761.8						N,6	368.8	573.6	983.3	1188.2	1393.1	1557.0
		N,7	C,1	573.6	942.4	1516.0					N,7	C,1	81.9	573.6	1638.9	1761.8		
			N,7	614.6	983.3	1229.2	1557.0	1638.9	1761.8			N,7	368.8	573.6	737.5	983.3	1557.0	
	C,2	C,3	C,2	532.6	1106.3	1352.1	1516.0	1761.8		C,2	C,3	C,2	81.9	573.6	1638.9	1761.8		
			C,3	614.6	983.3	1229.2	1557.0	1638.9	1761.8			C,3	368.8	573.6	737.5	983.3	1557.0	
		N,5	C,2	532.6	942.4	1188.2	1352.1				N,5	C,2	81.9	573.6	1638.9	1761.8		
			N,5	614.6	983.3	1229.2	1557.0	1638.9	1761.8			N,5	368.8	573.6	737.5	983.3	1557.0	
		N,6	C,2	632.6	942.4	1188.2	1516.0				N,6	C,2	81.9	573.6	1638.9	1761.8		
			N,6	614.6	983.3	1229.2	1557.0	1638.9	1761.8			N,6	368.8	573.6	737.5	983.3	1557.0	
	C,3	C,2	C,3	1188.2	1393.1	1516.0	1720.9			C,3	C,2	C,3	81.9	573.6	1638.9	1761.8		
			C,2	614.6	983.3	1229.2	1557.0	1638.9	1761.8			C,2	368.8	573.6	737.5	983.3	1557.0	
		C,4	C,3	696.5	1106.3	1393.1	1516.0	1761.8			C,4	C,3	81.9	573.6	1638.9	1761.8		
			C,4	614.6	983.3	1229.2	1557.0	1638.9	1761.8			C,4	368.8	573.6	737.5	983.3	1557.0	
	C,4	C,3	C,4	532.6	1106.3	1188.2	1352.1	1516.0	1761.8	C,4	C,3	C,4	81.9	573.6	1638.9	1761.8		
			C,3	614.6	983.3	1229.2	1557.0	1638.9	1761.8			C,3	368.8	573.6	737.5	983.3	1557.0	
		N,7	C,4	573.6	942.4	1516.0					N,7	C,4	81.9	573.6	1638.9	1761.8		
			N,7	614.6	983.3	1229.2	1557.0	1638.9	1761.8			N,7	368.8	573.6	737.5	983.3	1557.0	
	N,5	C,2	N,5	1188.2	1393.1	1516.0	1720.9			N,5	C,2	N,5	81.9	573.6	1638.9	1761.8		
			C,2	614.6	983.3	1229.2	1557.0	1638.9	1761.8			C,2	368.8	573.6	737.5	983.3	1557.0	
	N,6	C,1	N,6	532.6	1188.2	1516.0	1679.9			N,6	C,1	N,6	81.9	573.6	1638.9	1761.8		
			C,1	573.6	983.3	1229.2	1557.0	1761.8				C,1	368.8	573.6	737.5	983.3	1557.0	
		C,2	N,6	532.6	1188.2	1393.1	1516.0	1720.9			C,2	N,6	81.9	573.6	1638.9	1761.8		
			C,2	573.6	983.3	1229.2	1557.0	1761.8				C,2	368.8	573.6	737.5	983.3	1557.0	
	N,7	C,1	N,7	532.6	1188.2	1516.0	1679.9			N,7	C,1	N,7	81.9	573.6	1638.9	1761.8		
			C,1	614.6	983.3	1229.2	1557.0	1638.9	1761.8			C,1	368.8	573.6	737.5	983.3	1557.0	
		C,4	N,7	532.6	696.5	1393.1	1516.0	1761.8			C,4	N,7	81.9	573.6	1638.9	1761.8		
			C,4	614.6	983.3	1229.2	1557.0	1638.9	1761.8			C,4	368.8	573.6	737.5	983.3	1557.0	
	H,8	N,5	H,8	532.6	943.4	1188.2	1352.1	1720.9		H,8	N,5	H,8	81.9	573.6	1638.9	1761.8		
			N,5	614.6	983.3	1229.2	1557.0	1638.9	1761.8			N,5	368.8	573.6	737.5	983.3	1557.0	
	H,9	N,5	H,9	532.6	943.4	1188.2	1352.1	1720.9		H,9	N,5	H,9	81.9	573.6	1638.9	1761.8		
			N,5	614.6	983.3	1229.2	1557.0	1638.9	1761.8			N,5	368.8	573.6	737.5	983.3	1557.0	
	H,10	C,3	H,10	532.6	901.4	1106.3	1352.1	1516.0	1761.8	H,10	C,3	H,10	81.9	573.6	1638.9	1761.8		
			C,3	614.6	983.3	1229.2	1557.0	1638.9	1761.8			C,3	368.8	573.6	737.5	983.3	1557.0	
	H,11	C,4	H,11	532.6	696.5	1393.1	1516.0	1761.8		H,11	C,4	H,11	81.9	573.6	1638.9	1761.8		
			C,4	614.6	983.3	1229.2	1557.0	1638.9	1761.8			C,4	368.8	573.6	737.5	983.3	1557.0	
	O,12	C,1	O,12	532.6	1188.2	1516.0				O,12	C,1	O,12	81.9	573.6	1638.9	1761.8		
			C,1	81.9	573.6	1229.2	1638.9	1761.8				C,1	368.8	573.6	737.5	983.3	1557.0	

Column names for stretching (left columns) and bending (right columns) frequencies are in cm^{-1} units and as follows:

Base, Atom & num., Bonding atom, Reference atom, 1st mode, 2nd mode, 3rd mode, 4th mode, 5th mode, 6th mode are shown in 2 sets of columns.

Table 6. Frequencies of Thymine base bonds, highest intensities. Frequencies are in cm^{-1} .

Reaction Coordinate unit of [cm−1]

strech

base	atom & num	Versus atom	Ref atom	1th peak	2th peak	3th peak	4th peak	5th peak	6th peak
THY	C,1	N,11	C,1	901.4	1065.3	1188.2	1352.1		
			N,11	491.7	1229.2	1393.1	1597.9	1679.9	1761.8
		N,12	C,1	737.5	942.4	1352.1	1434.1	1720.9	
			N,12	1597.9	1761.8				
	C,2	C,3	C,2	655.6	1188.2	1311.1	1475.0	1720.9	1843.7
			C,3	1597.9	1761.8				
		N,12	C,2	737.5	942.4	1352.1	1434.1	1720.9	
			N,12	1597.9	1761.8				
	C,3	C,2	C,3	409.7	655.6	1024.3	1188.2		
			C,2	1597.9	1761.8				
		C,4	C,3	655.6	1065.3	1352.1			
			C,4	1597.9	1761.8				
		C,5	C,3	450.7	1065.3	1393.1	1761.8	2827.1	2950.0
			C,5	1597.9	1761.8				
	C,4	C,3	C,4	655.6	1188.2	1311.1	1475.0	1720.9	1843.7
			C,3	1597.9	1761.8				
		N,11	C,4	901.4	1065.3	1188.2	1352.1	1638.9	1720.9
			N,11	1597.9	1761.8				
	C,5	C,3	C,5	655.6	1188.2	1311.1	1475.0	1720.9	1843.7
			C,3	1597.9	1761.8				
	H,6	N,12	H,6	737.5	942.4	1352.1	1434.1	1720.9	
			N,12	1597.9	1761.8				
	H,7	C,4	H,7	655.6	1065.3	1352.1			
			C,4	1597.9	1761.8				
	H,8	C,5	H,8	450.7	1065.3	1393.1	1761.8	2827.1	2950.0
			C,5	1597.9	1761.8				
	H,9	C,5	H,9	450.7	1065.3	1393.1	1761.8	2827.1	2950.0
			C,5	1597.9	1761.8				
	H,10	C,5	H,10	450.7	1065.3	1393.1	1761.8	2827.1	2950.0
			C,5	1597.9	1761.8				
	N,11	C,1	N,11	1106.3	1352.1	1516.0	17209.0	2130.6	
			C,1	491.7	1229.2	1393.1	1597.9	1679.9	1761.8
		C,4	N,11	409.7	655.6	1024.3	1188.2		
			C,4	491.7	1229.2	1393.1	1597.9	1679.9	1761.8
	N,12	C,1	N,12	1106.3	1352.1	1516.0	17209.0	2130.6	
			C,1	1597.9	1761.8				
		C,2	N,12	409.7	655.6	1024.3	1188.2		
			C,2	1597.9	1761.8				
	O,13	C,1	O,13	1106.3	1720.9				
			C,1	491.7	1516.0	1679.9	1761.8		
	O,14	C,2	O,14	409.7	655.6	1024.3	1188.2	1352.1	1475.0
			C,2	491.7	1516.0	1679.9	1761.8		

bend

base	atom & num	Versus atom	Ref atom	1th peak	2th peak	3th peak	4th peak	5th peak
C,1	N,11	C,1	368.8	1393.1	1597.9	1761.8	1884.7	
		N,11	327.8	491.7	778.5	1065.3		
	N,12	C,1	368.8	1393.1	1597.9	1761.8	1884.7	
		N,12	368.8	942.4	1188.2	1311.1		
C,2	C,3	C,2	368.8	1393.1	1597.9	1761.8	1884.7	
		C,3	368.8	942.4	1188.2	1311.1		
	N,12	C,2	368.8	1393.1	1597.9	1761.8	1884.7	
		N,12	368.8	942.4	1188.2	1311.1		
C,3	C,2	C,3	368.8	1393.1	1597.9	1761.8	1884.7	
		C,2	368.8	942.4	1188.2	1311.1		
	C,4	C,3	368.8	1393.1	1597.9	1761.8	1884.7	
		C,4	368.8	942.4	1188.2	1311.1		
	CH3,5	C,3	368.8	1393.1	1597.9	1761.8	1884.7	
		C,5	368.8	942.4	1188.2	1311.1		
C,4	C,3	C,4	368.8	1393.1	1597.9	1761.8	1884.7	
		C,3	368.8	942.4	1188.2	1311.1		
	N,11	C,4	368.8	1393.1	1597.9	1761.8	1884.7	
		N,11	368.8	942.4	1188.2	1311.1		
C,5	C,3	C,5	368.8	1393.1	1597.9	1761.8	1884.7	
		C,3	1597.9	1761.8				
H,6	N,12	H,6	368.8	1393.1	1597.9	1761.8	1884.7	
		N,12	368.8	942.4	1188.2	1311.1		
H,7	C,4	H,7	655.6	1065.3	1352.1			
		C,4	368.8	942.4	1188.2	1311.1		
H,8	C,5	H,8	368.8	1393.1	1597.9	1761.8	1884.7	
		C,5	368.8	942.4	1188.2	1311.1		
H,9	C,5	H,9	368.8	1393.1	1597.9	1761.8	1884.7	
		C,5	1597.9	1761.8				
H,10	C,5	H,10	368.8	1393.1	1597.9	1761.8	1884.7	
		C,5	368.8	942.4	1188.2	1311.1		
N,11	C,1	N,11	368.8	1393.1	1597.9	1761.8	1884.7	
		C,1	368.8	942.4	1188.2	1311.1		
	C,4	N,11	368.8	1393.1	1597.9	1761.8	1884.7	
		C,4	368.8	942.4	1188.2	1311.1		
N,12	C,1	N,12	368.8	1393.1	1597.9	1761.8	1884.7	
		C,1	368.8	942.4	1188.2	1311.1		
	C,2	N,12	368.8	1393.1	1597.9	1761.8	1884.7	
		C,2	368.8	942.4	1188.2	1311.1		
O,13	C,1	O,13	368.8	1393.1	1597.9	1761.8	1884.7	
		C,1	368.8	942.4	1188.2	1311.1		
O,14	C,2	O,14	368.8	1393.1	1597.9	1761.8	1884.7	
		C,2	368.8	942.4	1188.2	1311.1		

Column names for stretching (left columns) and bending (right columns) frequencies are in cm^{-1} units and as follows:

Base, Atom & num., Bonding atom, Reference atom, 1st mode, 2nd mode, 3rd mode, 4th mode, 5th mode, 6th mode are shown in 2 sets of columns.

Table 7. Frequencies of Adenine base bonds, highest intensities. Frequencies are in cm^{-1} .

Reaction Coordinate unit of [cm−1]	strech									bend								
base	atom & num	Versus atom	Ref atom	1th peak	2th peak	3th peak	4th peak	5th peak	6th peak	base	atom & num	Versus atom	Ref atom	1th peak	2th peak	3th peak	4th peak	5th peak
ADE	C,1	C,2	C,1	532.6	1106.3	1557.0	1884.8	2171.7		C,1	C,2	C,1	491.7	819.5	1147.2	1597.9	1720.9	
			C,2	532.6	655.6	1393.1	1597.9	1884.8				C,2	327.8	573.6	819.5	1270.2		
		N,11	C,1	327.8	532.6	655.6	819.5	1393.1	1720.9		N,11	C,1	491.7	819.5	1147.2	1597.9	1720.9	
			N,11	491.7	655.6	1393.1	1557.0	1884.8				N,11	327.8	655.6	983.3	1147.2	1557.0	1720.9
		N,13	C,1	532.6	819.5	983.3	1147.2	1393.1			N,13	C,1	491.7	819.5	1147.2	1597.9	1720.9	
			N,13	81.9	368.8	655.6	819.5	1434.1	1557.0			N,13	327.8	573.6	655.6	819.5	1393.1	
	C,2	C,1	C,2	532.6	1393.1	1557.0				C,2	C,1	C,2	491.7	819.5	1147.2	1597.9	1720.9	
			C,1	532.6	655.6	1147.2	1393.1	1597.9	1884.8			C,1	327.8	573.6	655.6	819.5	1393.1	
		C,3	C,2	532.6	1393.1	1557.0					C,3	C,2	491.7	819.5	1147.2	1597.9	1720.9	
			C,3	532.6	655.6	1147.2	1393.1	1597.9				C,3	327.8	573.6	655.6	819.5	1393.1	
		N,12	C,2	327.8	778.5	1147.2	1270.2	1393.1			N,12	C,2	491.7	819.5	1147.2	1597.9	1720.9	
			N,12	532.6	655.6	1147.2	1393.1	1597.9				N,12	327.8	573.6	655.6	819.5	1393.1	
	C,3	C,2	C,3	532.6	1106.3	1557.0	1884.8	2171.7		C,3	C,2	C,3	491.7	819.5	1147.2	1597.9	1720.9	
			C,2	532.6	655.6	1147.2	1393.1	1597.9	1884.8			C,2	327.8	573.6	655.6	819.5	1393.1	
		N,10	C,3	532.6	819.5	1147.2	1311.1	1597.9			N,10	C,3	491.7	819.5	1147.2	1597.9	1720.9	
			N,10	532.6	655.6	1147.2	1393.1	1597.9	1884.8			N,10	327.8	573.6	655.6	819.5	1393.1	
		N,14	C,3	491.7	614.6	819.5	1147.2	1557.0			N,14	C,3	81.9	655.6	819.5	1147.2	1434.1	1597.9
			N,14	532.6	655.6	1147.2	1393.1	1597.9	1884.8			N,14	327.8	573.6	655.6	819.5	1393.1	
	C,4	N,10	C,4	532.6	819.5	1147.2	1311.1	1597.9		C,4	N,10	C,4	81.9	655.6	819.5	1147.2	1434.1	1597.9
			N,10	532.6	655.6	1147.2	1393.1	1597.9	1884.8			N,10	327.8	573.6	655.6	819.5	1393.1	
		N,12	C,4	327.8	778.5	1147.2	1270.2	1393.1			N,12	C,4	81.9	655.6	819.5	1147.2	1434.1	1597.9
			N,12	532.6	655.6	1147.2	1393.1	1597.9	1884.8			N,12	327.8	573.6	655.6	819.5	1393.1	
	C,5	N,13	C,5	532.6	819.5	983.3	1147.2	1393.1		C,5	N,13	C,5	81.9	655.6	819.5	1147.2	1434.1	1597.9
			N,13	532.6	655.6	1147.2	1393.1	1597.9	1884.8			N,13	327.8	573.6	655.6	819.5	1393.1	
		N,14	C,5	491.7	614.6	819.5	1147.2	1270.2	1557.0		N,14	C,5	491.7	819.5	1147.2	1597.9	1720.9	
			N,14	532.6	655.6	1147.2	1393.1	1597.9	1884.8			N,14	327.8	573.6	655.6	819.5	1393.1	
	H,6	N,11	H,6	327.8	532.6	655.6	819.5	1393.1	1720.9	H,6	N,11	H,6	491.7	819.5	1147.2	1597.9	1720.9	
			N,11	532.6	655.6	1147.2	1393.1	1597.9	1884.8			N,11	327.8	573.6	655.6	819.5	1393.1	
	H,7	N,11	H,7	327.8	532.6	655.6	819.5	1393.1	1720.9	H,7	N,11	H,7	491.7	819.5	1147.2	1597.9	1720.9	
			N,11	532.6	655.6	1147.2	1393.1	1597.9	1884.8			N,11	327.8	573.6	655.6	819.5	1393.1	
	H,8	C,5	H,8	491.7	614.6	778.5	1393.1	1597.9	1720.9	H,8	C,5	H,8	491.7	819.5	1147.2	1597.9	1720.9	
			C,5	532.6	655.6	1147.2	1393.1	1597.9	1884.8			C,5	327.8	573.6	655.6	819.5	1393.1	
	H,9	C,4	H,9	327.8	614.6	819.5	1106.3	1393.1	1679.9	H,9	C,4	H,9	491.7	819.5	1147.2	1597.9	1720.9	
			C,4	532.6	655.6	1147.2	1393.1	1597.9	1884.8			C,4	327.8	573.6	655.6	819.5	1393.1	
	N,10	C3	N,10	532.6	1393.1	1557.0				N,10	C3	N,10	491.7	819.5	1147.2	1597.9	1720.9	
			C,3	532.6	655.6	1147.2	1393.1	1597.9	1884.8			C,3	327.8	573.6	655.6	819.5	1393.1	
		C,4	N,10	327.8	614.6	819.5	1106.3	1393.1	1679.9		C,4	N,10	491.7	819.5	1147.2	1597.9	1720.9	
			C,4	532.6	655.6	1147.2	1393.1	1597.9	1884.8			C,4	327.8	573.6	655.6	819.5	1393.1	
	N,11	C,1	N,11	532.6	614.6	1393.1	1557.0	1884.8		N,11	C,1	N,11	491.7	819.5	1147.2	1597.9	1720.9	
			C,1	327.8	491.7	655.6	1393.1	1557.0	1884.8			C,1	327.8	573.6	655.6	819.5	1393.1	
	N,12	C,2	N,12	532.6	614.6	1393.1	1557.0	1884.8		N,12	C,2	N,12	491.7	819.5	1147.2	1597.9	1720.9	
			C,2	327.8	491.7	655.6	1393.1	1557.0	1884.8			C,2	327.8	573.6	655.6	819.5	1393.1	
		C,4	N,12	327.8	614.6	819.5	1106.3	1393.1	1679.9		C,4	N,12	491.7	819.5	1147.2	1597.9	1720.9	
			C,4	327.8	491.7	655.6	1393.1	1557.0	1884.8			C,4	327.8	573.6	655.6	819.5	1393.1	
	N,13	C,1	N,13	532.6	1393.1	1557.0				N,13	C,1	N,13	491.7	819.5	1147.2	1597.9	1720.9	
			C,1	81.9	368.8	655.6	819.5	1434.1	1557.0			C,1	327.8	573.6	655.6	819.5	1393.1	
		C,5	N,13	491.7	614.6	819.5	1393.1	1597.9	1720.9		C,5	N,13	491.7	819.5	1147.2	1597.9	1720.9	
			C,5	81.9	368.8	655.6	819.5	1434.1	1557.0			C,5	327.8	573.6	655.6	819.5	1393.1	
	N,14	C,3	N,14	532.6	1393.1	1557.0				N,14	C,3	N,14	81.9	655.6	819.5	1147.2	1434.1	1597.9
			C,3	81.9	368.8	655.6	819.5	1434.1	1557.0			C,3	327.8	573.6	655.6	819.5	1393.1	
		C,5	N,14	491.7	614.6	819.5	1393.1	1597.9	1720.9		C,5	N,14	491.7	819.5	1147.2	1597.9	1720.9	
			C,5	81.9	368.8	655.6	819.5	1434.1	1557.0			C,5	327.8	573.6	655.6	819.5	1393.1	

Column names for stretching (left columns) and bending (right columns) frequencies are in cm^{-1} units and as follows:

Base, Atom & num., Bonding atom, Reference atom, 1st mode, 2nd mode, 3rd mode, 4th mode, 5th mode, 6th mode are shown in 2 sets of columns.

Table 8. Frequencies of Guanine base bonds, highest intensities. Frequencies are in cm^{-1} .

Reaction Coordinate unit of [cm−1]

strech

base	atom & num	Versus atom	Ref atom	1th peak	2th peak	3th peak	4th peak	5th peak	6th peak
GUA	C,1	C,3	C,1	737.5	860.4	1106.3	1188.2	1352.1	2048.6
			C,3	614.6	860.4	1188.2	1352.1	1557.0	1638.9
		N,12	C,1	532.6	860.4	983.3			
			N,12	81.9	614.6	778.5	1229.2	1352.1	1557.0
	C,2	N,11	C,2	450.7	573.6	860.4	1311.1		
			N,11	81.9	614.6	778.5	1229.2	1352.1	1557.0
		N,12	C,2	532.6	860.4	983.3			
			N,12	81.9	614.6	778.5	1229.2	1352.1	1557.0
		N,14	C,2	573.6	860.4	983.3	1188.2	1638.9	2253.5
			N,14	81.9	614.6	778.5	1229.2	1352.1	1557.0
	C,3	C,1	C,3	614.6	901.4	1352.1	1516.0	1638.9	1802.8
			C,1	614.6	778.5	860.4	1188.2	1352.1	1557.0
		C,4	C,3	737.5	860.4	1557.0	1638.9	2253.5	
			C,4	614.6	778.5	860.4	1188.2	1352.1	1557.0
		N,13	C,3	737.5	819.5	1352.1	1679.9		
			N,13	614.6	778.5	860.4	1188.2	1352.1	1557.0
	C,4	C,3	C,4	327.8	573.6	860.4	1106.3	1188.2	
			C,3	614.6	778.5	860.4	1188.2	1352.1	1557.0
		N,10	C,4	737.5	860.4	1229.2			
			N,10	614.6	778.5	860.4	1188.2	1352.1	1557.0
		N,14	C,4	573.6	860.4	983.3			
			N,14	614.6	778.5	860.4	1188.2	1352.1	1557.0
	C,5	N,10	C,5	737.5	860.4	1229.2			
			N,10	614.6	778.5	860.4	1188.2	1352.1	1557.0
		N,13	C,5	737.5	819.5	1352.1	1679.9		
			N,13	614.6	778.5	860.4	1188.2	1352.1	1557.0
	H,6	N,12	H,6	532.6	860.4	983.3			
			N,12	614.6	778.5	860.4	1188.2	1352.1	1557.0
	H,7	N,11	H,7	450.7	573.6	778.5	860.4	1311.1	
			N,11	614.6	778.5	860.4	1188.2	1352.1	1557.0
	H,8	N,11	H,8	450.7	573.6	778.5	860.4	1311.1	
			N,11	614.6	778.5	860.4	1188.2	1352.1	1557.0
	H,9	C,5	H,9	737.5	860.4	1229.2	1557.0	1679.9	
			C,5	614.6	778.5	860.4	1188.2	1352.1	1557.0
	N,10	C,4	N,10	737.5	860.4	1557.0	1638.9	2253.5	
			C,4	614.6	778.5	860.4	1188.2	1352.1	1557.0
		C,5	N,10	737.5	860.4	1229.2	1557.0	1679.9	
			C,5	614.6	778.5	860.4	1188.2	1352.1	1557.0
	N,11	C,2	N,11	532.6	1557.0				
			C,2	614.6	778.5	860.4	1188.2	1352.1	1557.0
	N,12	C,1	N,12	614.6	1352.1	1516.0	1638.9	1802.8	
			C,1	81.9	614.6	778.5	1229.2	1352.1	1557.0
		C,2	N,12	532.6	1188.2	1311.1	1557.0	1761.8	
			C,2	81.9	614.6	778.5	1229.2	1352.1	1557.0
	N,13	C,3	N,13	737.5	860.4	1106.3	1188.2	1352.1	
			C,3	81.9	614.6	778.5	1229.2	1352.1	1557.0
		C,5	N,13	737.5	860.4	1229.2	1557.0	1679.9	
			C,5	81.9	614.6	778.5	1229.2	1352.1	1557.0
	N,14	C,2	N,14	532.6	1188.2	1311.1	1557.0	1761.8	
			C,2	81.9	614.6	778.5	1229.2	1352.1	1557.0
		C,4	N,14	737.5	860.4	1557.0	1638.9	2253.5	
			C,4	81.9	614.6	778.5	1229.2	1352.1	1557.0
	O,15	C,1	O,15	614.6	737.5	1352.1	1516.0	1638.9	
			C,1	368.8	614.6	1557.0	1679.9		

bend

base	atom & num	Versus atom	Ref atom	1th peak	2th peak	3th peak	4th peak	5th peak
C,1	C,3	C,1	368.8	614.6	1557.0	1679.9		
		C,3	327.8	614.6	737.5	1229.2		
	N,12	C,1	368.8	614.6	1557.0	1679.9		
		N,12	614.6	737.5	860.4	1270.2		
C,2	N,11	C,2	368.8	614.6	1557.0	1679.9		
		N,11	614.6	737.5	860.4	1270.2		
	N,12	C,2	368.8	614.6	1557.0	1679.9		
		N,12	614.6	737.5	860.4	1270.2		
	N,14	C,2	368.8	614.6	1557.0	1679.9		
		N,14	614.6	737.5	860.4	1270.2		
C,3	C,1	C,3	368.8	614.6	1557.0	1679.9		
		C,1	614.6	737.5	860.4	1270.2		
	C,4	C,3	368.8	614.6	1557.0	1679.9		
		C,4	614.6	737.5	860.4	1270.2		
	N,13	C,3	368.8	614.6	1557.0	1679.9		
		N,13	614.6	737.5	860.4	1270.2		
C,4	C,3	C,4	368.8	614.6	1557.0	1679.9		
		C,3	614.6	737.5	860.4	1270.2		
	N,10	C,4	368.8	614.6	1557.0	1679.9		
		N,10	614.6	737.5	860.4	1270.2		
	N,14	C,4	368.8	614.6	1557.0	1679.9		
		N,14	614.6	737.5	860.4	1270.2		
C,5	N,10	C,5	368.8	614.6	1557.0	1679.9		
		N,10	614.6	737.5	860.4	1270.2		
	N,13	C,5	368.8	614.6	1557.0	1679.9		
		N,13	614.6	737.5	860.4	1270.2		
H,6	N,12	H,6	368.8	614.6	1557.0	1679.9		
		N,12	614.6	737.5	860.4	1270.2		
H,7	N,11	H,7	368.8	614.6	1557.0	1679.9		
		N,11	614.6	737.5	860.4	1270.2		
H,8	N,11	H,8	368.8	614.6	1557.0	1679.9		
		N,11	614.6	737.5	860.4	1270.2		
H,9	C,5	H,9	368.8	614.6	1557.0	1679.9		
		C,5	614.6	737.5	860.4	1270.2		
N,10	C,4	N,10	368.8	614.6	1557.0	1679.9		
		C,4	614.6	737.5	860.4	1270.2		
	C,5	N,10	368.8	614.6	1557.0	1679.9		
		C,5	614.6	737.5	860.4	1270.2		
N,11	C,2	N,11	368.8	614.6	1557.0	1679.9		
		C,2	614.6	737.5	860.4	1270.2		
N,12	C,1	N,12	368.8	614.6	1557.0	1679.9		
		C,1	614.6	737.5	860.4	1270.2		
	C,2	N,12	368.8	614.6	1557.0	1679.9		
		C,2	614.6	737.5	860.4	1270.2		
N,13	C,3	N,13	368.8	614.6	1557.0	1679.9		
		C,3	614.6	737.5	860.4	1270.2		
	C,5	N,13	368.8	614.6	1557.0	1679.9		
		C,5	614.6	737.5	860.4	1270.2		
N,14	C,2	N,14	368.8	614.6	1557.0	1679.9		
		C,2	614.6	737.5	860.4	1270.2		
	C,4	N,14	368.8	614.6	1557.0	1679.9		
		C,4	614.6	737.5	860.4	1270.2		
O,15	C,1	O,15	368.8	614.6	1557.0	1679.9		
		C,1	614.6	737.5	860.4	1270.2		

Column names for stretching (left columns) and bending (right columns) frequencies are in cm^{-1} units and as follows:

Base, Atom & num., Bonding atom, Reference atom, 1st mode, 2nd mode, 3rd mode, 4th mode, 5th mode, 6th mode are shown in 2 sets of columns.

stretch [cm^{-1}]										stretch [cm^{-1}]								
base	atom & num	Versus atom	Ref. atom	1st peak	2nd peak	3rd peak	4th peak	5th peak	6th peak	atom & num	Versus atom	Ref. atom	1st peak	2nd peak	3rd peak	4th peak	5th peak	6th peak

strech								
atom & num	Versus atom	Ref atom	1th peak	2th peak	3th peak	4th peak	5th peak	6th peak

bend							
atom & num	Versus atom	Ref atom	1th peak	2th peak	3th peak	4th peak	5th peak
